# Effect of Chiral Arm Angle on the Stiffness, Auxeticity and Energy Absorption of Polyamide-12 Tetra-Chiral Lattices Under Quasi-Static Compression: A Numerical Baseline Study

**DOI:** 10.3390/polym18192427

**Published:** 2026-10-05

**Authors:** Volkan Arıkan, Cenk Yanen

**Affiliations:** 1 Department of Mechanical Engineering, Osmaniye Korkut Ata University, 80000 Osmaniye, Türkiye; 2 Department of Mechanical Engineering, Faculty of Engineering, Firat University, 23119 Elazığ, Türkiye; cyanen@firat.edu.tr

**Keywords:** auxetic metamaterial, tetra-chiral lattice, polyamide-12, negative Poisson’s ratio, finite element analysis, collapse-mode bifurcation

## Abstract

Chiral auxetic lattices convert axial loading into node rotation, producing negative Poisson’s ratios and high energy absorption. This purely numerical study establishes a quasi-static baseline for additively manufacturable polyamide-12 (PA12) tetra-chiral lattices in which the chiral arm angle (0–90°) is treated as a design variable; no experiments were performed, and the model is verified rather than validated. Geometrically non-linear elastoplastic finite element analyses with a data-sheet-derived bilinear PA12 card and frictional self-contact were carried to 20% nominal strain and supported by mesh convergence, seeded-imperfection and friction sensitivity studies, a full-field lateral-strain measure and a refined sweep at 5° resolution. The arm angle switches the response between a stretch-dominated non-auxetic regime (0°, 50–90°) and a rotation-dominated auxetic regime (15–45°) whose lateral kinematics are not sensitive to mesh density, friction and seeded imperfections. The 15° lattice maximizes stiffness and peak force but is an isolated spike in the refined sweep, exhibits a collapse-mode bifurcation whose branch is selected by the discretization, and reaches the PA12 ductility limit after 3.5 mm of stroke. The 30° architecture sustains 9.4 mm before fracture onset and delivers the highest damage-bounded specific energy absorption of the auxetic window (820 J/kg on the baseline mesh), emerging as the design optimum.

## 1. Introduction

Mechanical metamaterials derive their effective behavior from engineered internal architecture rather than from chemical composition, and among them auxetic materials—solids exhibiting a negative Poisson’s ratio (NPR)—occupy a prominent position [1,2]. When compressed, an auxetic solid contracts laterally, drawing material into the loaded region instead of expelling it. This counter-intuitive kinematics produces a suite of functional advantages that has been documented extensively: elevated indentation and shear resistance, synclastic (dome-like) curvature under bending, enhanced fracture toughness and elevated energy absorption per unit mass [3,4,5]. These attributes have been surveyed for cellular auxetics in general [6] and have motivated interest in energy-absorbing and wearable applications [7], although the great majority of such applications are governed by dynamic loading and therefore lie outside the quasi-static scope of the present work. Polymeric realizations are particularly attractive because additive manufacturing (AM) makes complex auxetic cells directly printable, and a substantial body of work now addresses polymeric auxetic structures produced by fused filament fabrication and powder bed fusion, including their crashworthiness [8], cyclic response [9], finite-element-based deformation analysis of biopolymer cells [10] and machine-learning-assisted energy-absorption optimization [11]; the governing influence of internal cell architecture on the compressive and flexural response of printed polymer cores has likewise been quantified for hybrid sandwich configurations [12]; recent reviews have summarized both the fabrication routes [13] and the large-deformation energy absorption of additively manufactured auxetics [5].

Within the auxetic family, chiral cellular topologies form a distinct class in which straight ligaments are attached tangentially to central cylinders (nodes). Under remote loading, the eccentric ligament–node connection generates a couple on each node; the resulting node rotation winds or unwinds the attached ligaments, causing the cell to fold inward and yielding Poisson’s ratios approaching −1, as first analyzed by Prall and Lakes for the hexa-chiral honeycomb [14]. Subsequent theoretical and experimental work established the elastic constants of tri-, tetra- and hexa-chiral lattices and their anti-chiral counterparts [15,16,17,18,19,20], demonstrating that chiral honeycombs preserve their auxeticity over a much wider strain range than re-entrant topologies because rotation, rather than strut re-alignment, is the dominant deformation mechanism [14,16]. Three-dimensional chiral solids exploiting the same rotational kinematics have been shown to amplify the auxetic effect still further [21,22]. The tetra-chiral lattice—four ligaments per node arranged with rotational symmetry—is of particular engineering interest because it combines NPR behavior with orthogonal load paths compatible with rectangular structural envelopes [18,19]; chiral lattices have also been explored as polymer-based hybrid metamaterials with filled cells [23].

Polyamide-12 (PA12) has become the reference polymer for such studies. It is the dominant powder bed fusion feedstock, it is ductile enough to sustain the large cell-wall rotations that chiral collapse demands, and its elastoplastic response has been characterized in detail for both selective laser sintering and Multi Jet Fusion (MJF) routes [24,25]. Critically for the present work, Alomarah et al. [25] fabricated tetra-chiral and anti-tetra-chiral honeycombs from MJF PA12 and reported both experimental compression data and validated finite element models, providing a directly comparable benchmark for numerical studies of PA12 chiral lattices.

Despite this progress, a systematic gap remains. In virtually all published tetra-chiral studies the ligaments are taken either tangent to the nodes in the canonical configuration or normal to them in the anti-chiral variant; the angular orientation of the chiral arms with respect to the global loading axes is fixed by construction. Yet this angle directly controls the moment arm through which an applied load rotates the node, and therefore governs the balance between ligament stretching, ligament bending and node rotation—the three mechanisms that determine stiffness, auxeticity and energy dissipation. A parametric treatment of the arm angle across its full range, combined with displacement amplitudes deep in the geometrically and materially non-linear regime, is not available for polymeric chiral lattices. The few angle-related studies address ligament inclination in modified anti-chiral or hybrid cells at small strain [26,27]; comprehensive reviews of chiral metamaterial design confirm that the arm orientation is seldom varied systematically [28], the in-plane compression studies closest to the present configuration keep it fixed [29], and parameter-dependency studies in polymeric auxetics have so far concentrated on re-entrant families [8,30].

The present work addresses this gap through a parametric, geometrically and materially non-linear finite element campaign on PA12 tetra-chiral lattices whose chiral arm angle *θ* is varied from 0° to 90° in 15° increments inside a constant 50 mm× 50 mm × 50 mm envelope, subjected to quasi-static uniaxial compression of 5 mm and 10 mm (10% and 20% nominal strain), and refined to 5° resolution between 20° and 55°. The study is deliberately scoped as a purely numerical, quasi-static baseline: it maps the structural mechanics of the architecture free of rate and inertial effects and is intended as the reference layer on which rate-dependent models and experiments can subsequently be built, not as a design guide for impact-rated hardware. The specific objectives are: (i) to quantify the influence of *θ* on the effective Poisson’s ratio, stiffness, stress distribution and reaction force; (ii) to contrast the moderately non-linear response at 10% strain with the plasticity-dominated response at 20% strain; (iii) to derive mass- and density-normalized performance metrics and to complement the intact-material values with damage-bounded projections based on the measured elongation at break of PA12; (iv) to establish through mesh convergence, imperfection, friction and full-field kinematic checks which conclusions are numerically robust; and (v) to identify the mechanistic origin of the observed optima and translate them into design guidance for printable energy-absorbing components. To the authors’ knowledge, a systematic treatment of the tetra-chiral arm angle across the full 0–90° range under large plastic deformation has not been reported for a polymeric system; the closest studies vary ligament inclination in modified anti-chiral or hybrid cells at small strain [26,27].

## 2. Materials and Methods

### 2.1. Parametric Geometry

**Figure 1:**
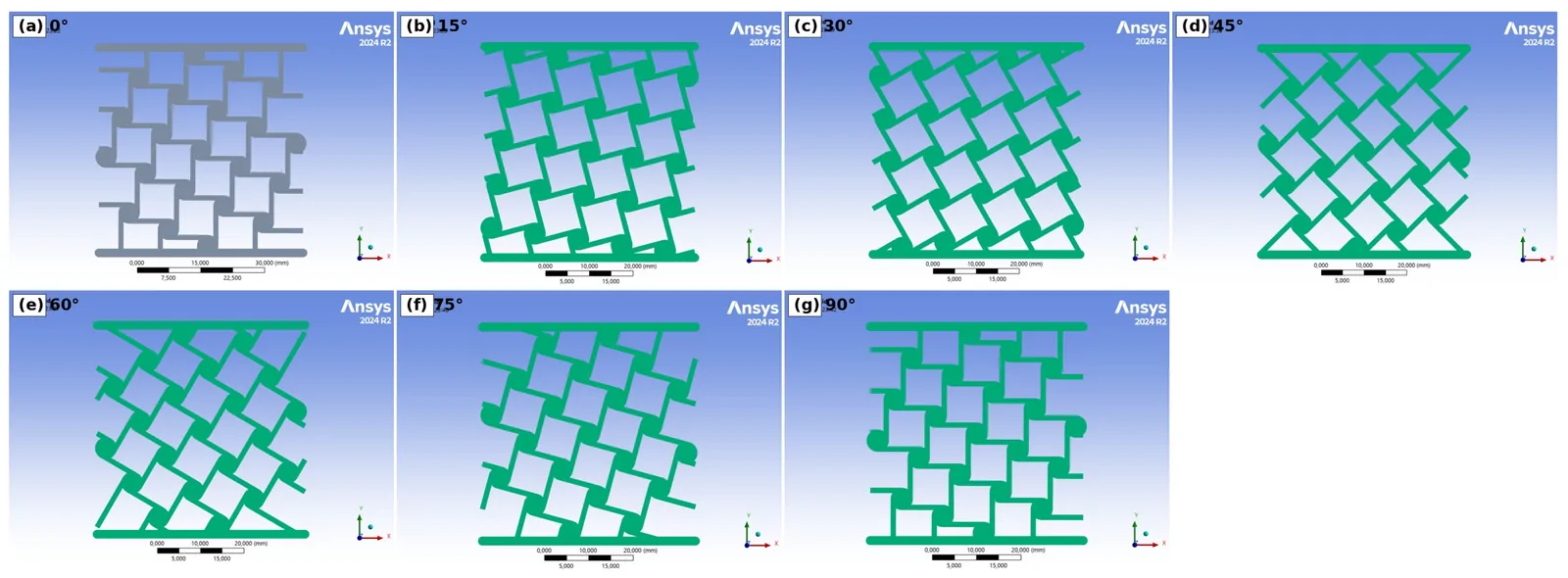
Geometric design of the tetra-chiral lattices with chiral arm angles *θ* = 0–90° in 15° increments (**a**–**g**). All models share the 50 × 50 × 50 mm envelope, node radius *r* = 5 mm, ligament length *L* = 10 mm and strut thickness *t* = 1.2 mm.

The parametric model family is shown in Figure 1. Each lattice occupies a bounding box of 50 mm × 50 mm × 50 mm and is built from tetra-chiral unit cells consisting of circular nodes of radius *r* = 5 mm connected by straight ligaments (chiral arms) of length *L* = 10 mm and thickness *t* = 1.2 mm. Nodes are arranged on a square grid and the assembly is extruded 50 mm in the out-of-plane (Z) direction, producing a prismatic cellular solid bounded by top and bottom face plates of 2 mm thickness. The plates provide a continuous load-introduction surface and avoid prescribing displacements on the free ends of isolated ligaments. Because the node pitch (≈14.1 mm) is not an integer divisor of the envelope and the node network is inclined to it at all angles other than 0° and 90°, the cells do not form a rectangular array; the number of complete closed cells enclosed by the envelope, counted on the geometry, is 16 at 0° and 90°, equivalent to a 4 × 4 array; 20 at 15° and 30°; 18 at 45°; 16 at 60° and 75°. The single design variable is the chiral arm angle *θ*, the inclination of the ligaments with respect to the global X–Y axes, varied from 0° to 90° in 15° increments and refined in 5° steps between 20° and 55° (Section 3.7). At *θ* = 0° and 90° the ligaments align with the envelope axes and the eccentricity of the ligament–node connection is minimal; at intermediate angles the connection becomes increasingly tangential, activating the chiral rotation mechanism. Because the envelope is held fixed while *θ* rotates the ligament network, the number of complete cells, and with it the solid volume (43,612–50,531 mm^3^) and relative density (0.349–0.404), vary with angle (cell count and relative density correlate at *r* = 0.97). All performance metrics are therefore also reported in mass- and density-normalized form (Section 3.7). The 1.2 mm wall thickness lies within the resolution of powder bed fusion, so every member of the family is geometrically printable in PA12 without topological modification [24,25].

### 2.2. Material Model and Its Scope

**Figure 2:**
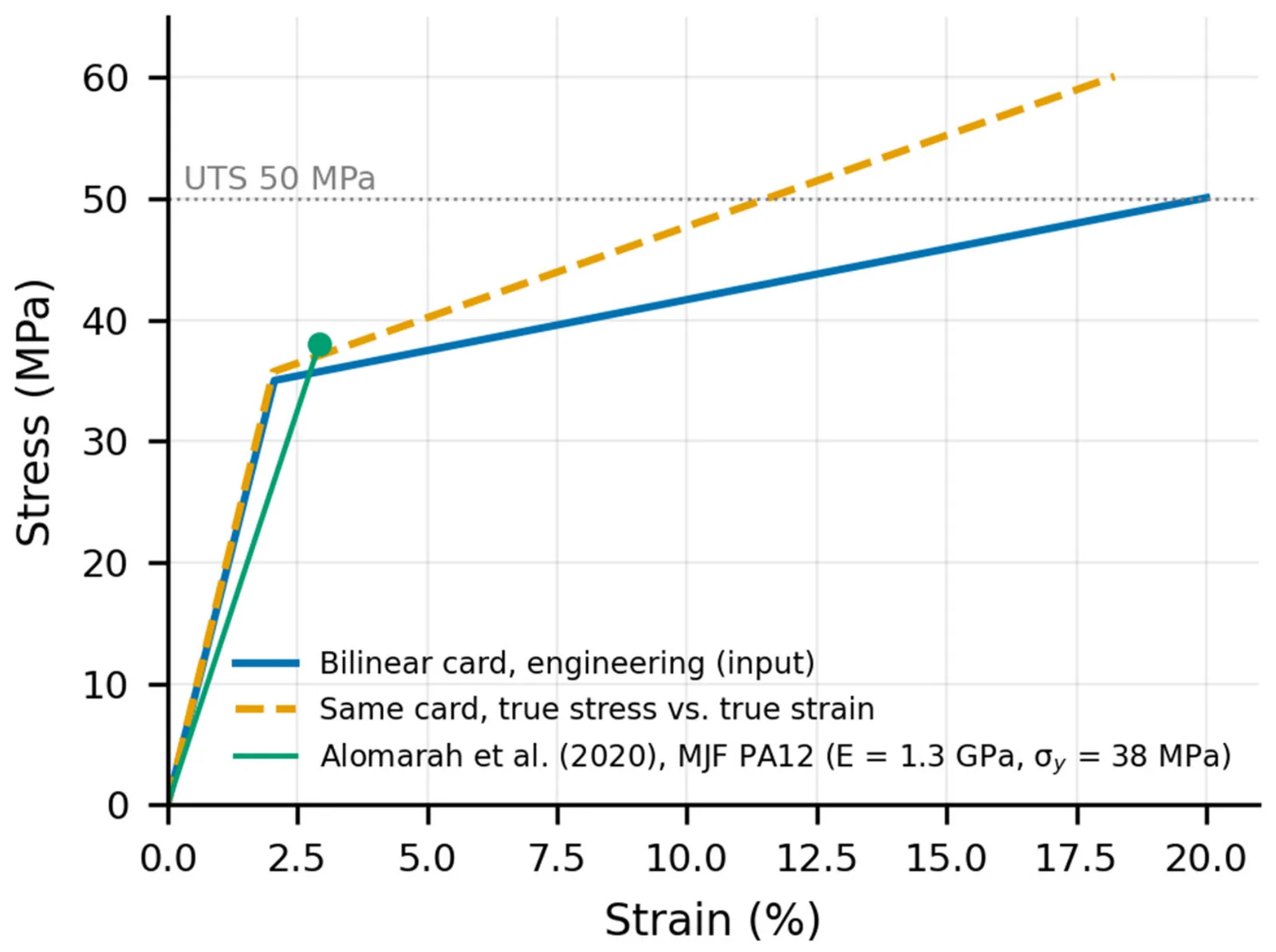
Stress–strain representation of the PA12 card: bilinear engineering input (solid), the same card expressed in true stress–true strain (dashed) and the measured modulus and yield point of MJF PA12 reported by Alomarah et al. [25]. The dotted line marks the 50 MPa data-sheet ultimate tensile strength.

All lattices were assigned polyamide-12 with a bilinear isotropic hardening (BISO) description; the parameters, which are those entered in Ansys, are listed in Table 1 and plotted in Figure 2. The elastic modulus, tensile strength, elongation at break and density were taken from the manufacturer’s data sheet for laser-sintered PA12 (EOS PA 2200, ISO 527-1/-2) [31,32]; a measured true stress–plastic strain curve of the specific powder batch was not available. The yield strength and tangent modulus were obtained by fitting a two-segment approximation between the yield knee and the ultimate point in engineering stress–strain space: with *σ_y_* = 35 MPa the yield strain is *ε_y_* = *σ_y_*/*E* = 2.06%, and the hardening slope follows as *E_t_* = (*σ_UTS_* − *σ_y_*)/( *ε_break_* − *ε_y_*) = (50 − 35)/(0.20 − 0.0206) ≈ 84 MPa. Because the BISO formulation interprets yield stress and tangent modulus as true-stress quantities, the same ultimate point corresponds in true stress–logarithmic plastic strain space to 60.1 MPa at a plastic strain of 0.147, and the slope that would reproduce it there is ≈165 MPa (dashed line in Figure 2). The adopted 84 MPa is therefore a conservative (soft) description of the post-yield response, and the force and energy predictions are correspondingly on the low side of what the data-sheet properties imply. A card calibrated on a measured true stress–plastic strain curve of the feedstock, or failing that a literature-based true-stress card such as the plastic table of [25], would be preferable and would raise the post-yield forces and energies; the angle ranking, which Section 3.6 shows to be governed by kinematics rather than by the hardening level, is not expected to change.

The card is consistent with independent characterizations of AM PA12 [24,25]: Alomarah et al. [25] measured *E* = 1.3 GPa, a 0.2% offset yield strength of 38 MPa, *ν* = 0.33 and *ρ* = 1000 kg/m^3^. The present values (*E* = 1700 MPa, *σ_y_* = 35 MPa, *ν* = 0.40, *ρ* = 930 kg/m^3^) bracket those measurements, the yield strengths agreeing to within 8%; the differences in modulus and density reflect the distinct consolidation of laser sintering versus MJF. The adopted hardening slope (84 MPa) is more conservative than both the true-stress equivalent of the data-sheet ultimate point (≈165 MPa) and the average slope implied by the plastic table of [25] (≈152 MPa between *ε_pl_* = 0 and 0.12), so the energy predictions are, in this respect, on the safe side. Calibration against a measured true stress–plastic strain curve of the actual feedstock is part of the experimental program of Section 4.5.

Two limitations of the constitutive description are stated at the outset. First, it is rate-independent, so the viscoelastic and strain-rate-hardening character of PA12 is not represented; this restricts all conclusions to quasi-static loading (Section 4.6). Second, it contains no damage or fracture model, so absolute energy values obtained with it are upper bounds for an idealized, fully ductile solid. The same simplification was adopted in the validated PA12 chiral-lattice models of Alomarah et al., who state explicitly that fracture was not considered [25]. To keep the reported performance figures physically meaningful without adding computational load, a post-processing damage-bounded projection is introduced in Section 2.5, exploiting the fact that PA12 has a well-defined elongation at break.

**Table 1:** Constitutive properties of the polyamide-12 (PA12) material model.

Property	Value	Source
Density, *ρ* (kg/m^3^)	930	EOS PA 2200 data sheet
Young’s modulus, *E* (MPa)	1700	EOS PA 2200 data sheet (ISO 527-1/-2)
Poisson’s ratio, *ν*	0.40	Adopted for PA12
Yield strength, *σ_y_* (MPa)	35	Bilinear fit (cf. 38 MPa, ref. [25])
Tangent modulus, *E_t_* (MPa)	84	Bilinear fit to *σ_UTS_* = 50 MPa at *ε* = 0.20
Elongation at break (%)	20 (X/Y), 10 (Z)	EOS PA 2200 data sheet
Hardening law	Bilinear isotropic	—

### 2.3. Finite Element Model, Mesh and Boundary Conditions

Quasi-static compression was simulated in Ansys Workbench Mechanical (version 2024 R2, Ansys Inc., Canonsburg, PA, USA; Static Structural) with the large-deflection formulation activated, so that finite rotations of the nodes and post-buckling folding of the ligaments are fully captured. Frictional self-contact (Coulomb coefficient *μ* = 0.2, pure-penalty formulation, program-controlled normal stiffness, pinball radius 1 mm) was defined between ligament and node surfaces that can approach one another during folding, using program-generated contact pairs (0–30 depending on the geometry); *μ* = 0.2 is close to the *μ* = 0.25 measured for MJF PA12 by a tilted-plane test [25], and the sensitivity of the results to *μ* is examined in Section 3.6. The load was applied in a single step with automatic time stepping (initial 100, minimum 50, maximum 1000 substeps) and a sparse direct solver; converged results were written at 52 time points per configuration (61 for 75–10 mm, where the solver bisected once).

**Figure 3:**
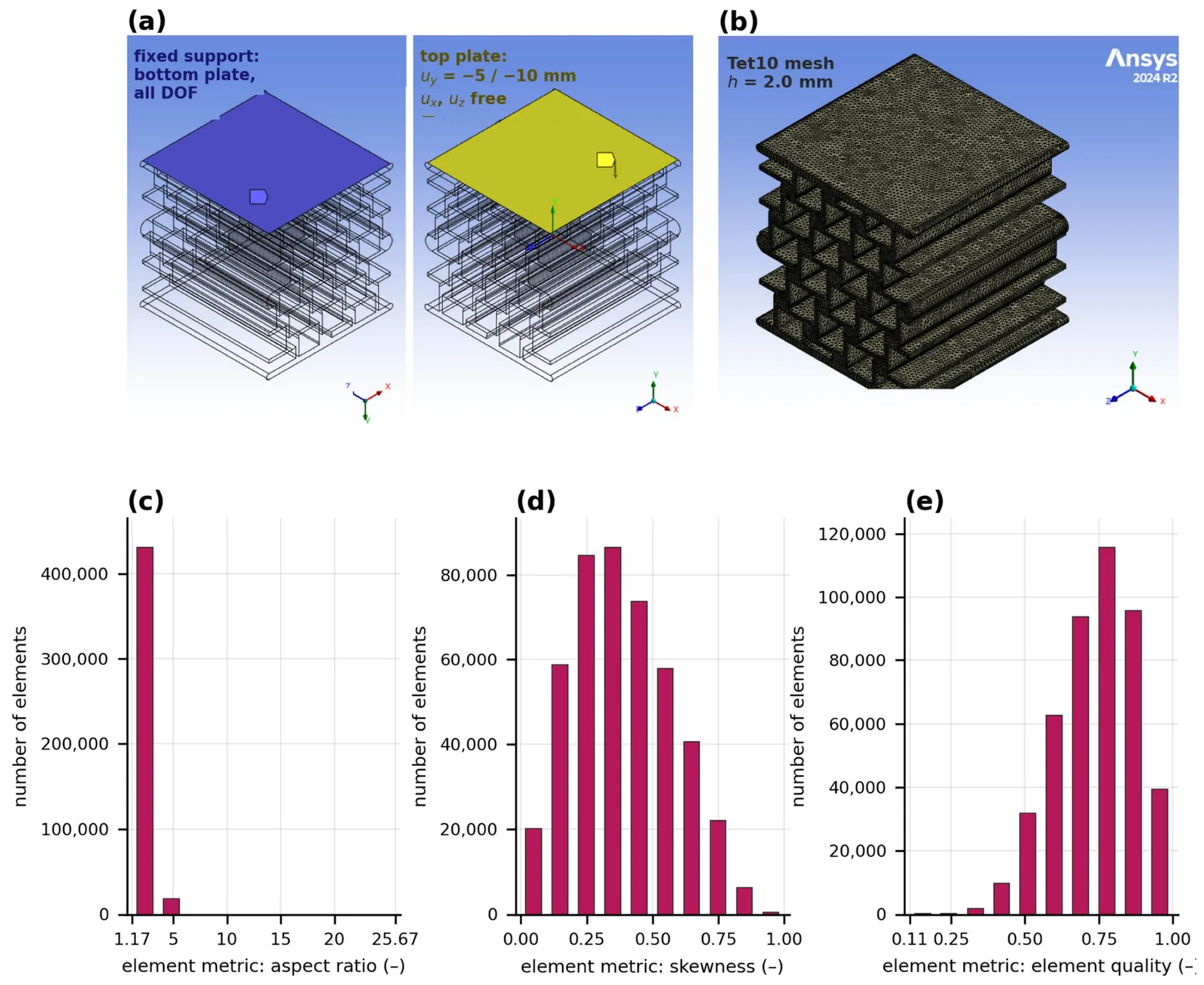
Analysis set-up: (**a**) fixed support on the bottom face and compressive displacement applied to the top face in −Y; (**b**) representative Tet10 mesh; (**c**) aspect-ratio; (**d**) skewness and (**e**) element-quality distributions of the mesh family.

The domains were discretized with quadratic ten-node tetrahedral elements (Tet10) generated with adaptive sizing at a global element size *h* = 2.0 mm, which places a single quadratic element through the 1.2 mm ligament thickness and about five elements along the 10 mm ligament. With the meshing method used, reducing the global element size does not change the through-thickness count; it increases the number of elements along the ligament and across its width. Quadratic elements are free of shear locking and reproduce the linear through-thickness strain of a bending ligament, so the discretization question that matters is the resolution of the plastic hinges along the ligament, addressed by the convergence study of Section 3.6. The baseline meshes comprised 52,336–75,498 nodes and 27,125–40,168 elements (Table 2). Mesh quality was verified through standard metrics (Figure 3c–e): the element-quality distribution peaks in the 0.75–0.88 band, skewness is concentrated below 0.5 (mode ≈ 0.25–0.38) and the aspect ratio of the overwhelming majority of elements is below 4.

**Table 2:** Geometry, mass, relative density, number of complete closed cells inside the 50 mm envelope and baseline mesh statistics (*h* = 2.0 mm) of the seven primary PA12 lattices.

θ (°)	Volume (mm^3^)	Mass (g)	ρ*_rel_* (–)	Complete Cells	Nodes	Elements
0	45,724	42.52	0.366	16	52,336	27,125
15	50,507	46.97	0.404	20	70,915	38,085
30	50,531	46.99	0.404	20	64,224	34,320
45	46,738	43.47	0.374	18	67,685	37,915
60	46,166	42.94	0.369	16	52,728	27,269
75	45,517	42.33	0.364	16	56,731	29,768
90	45,724	42.52	0.366	16	52,356	27,133

The boundary conditions are illustrated in Figure 3a. The bottom face plate was fully fixed, equivalent to a specimen bonded to a rigid, perfectly rough lower platen; the top face plate was driven by a prescribed displacement of −5 mm or −10 mm in the global Y direction with its X and Z translations left free, which corresponds to a frictionless upper platen. The rough lower boundary is stiffer than the lubricated platens of a standard compression test and suppresses lateral sliding of the bottom row of cells; its influence on the location of the first collapsing row is discussed in Section 3.2 and Section 4.5. Three named selections were used as kinematic probes: TOP_MID on the loaded face (verification of the imposed displacement), SIDE_MID at the mid-height of the lateral free surface (single-point lateral displacement *u_x_*) and FRONT_FACE, the complete lateral free surface normal to X, whose area-averaged X displacement provides the full-field lateral measure of Section 2.4. The reaction force was recorded at the displacement boundary condition throughout the loading history.

### 2.4. Derived Performance Metrics

Fourteen primary configurations (seven angles × two displacement amplitudes, labeled θ-δ, e.g., 15–10) and six configurations of the refined sweep (20°, 25°, 35°, 40°, 50°, 55° at 10 mm) were post-processed at the end of loading (*t* = 1 s). From the primary outputs (maximum von Mises stress *σ_vm_*, maximum principal stress *σ*_1_, reaction force *F*(*t*), total strain energy *E_abs_*, external work *W_ext_* = ∫F∗dδ, solid volume *V* and mass *m*) the following metrics were computed:

(1) Relative density: ρ*_rel_* = *V* / *V_box_*, with *V_box_* = 125,000 mm^3^;
(2) Local effective Poisson’s ratio: *ν_eff_* = −*ε_x_*/*ε_y_* = *u_x_*/δ, where *ε_x_* = *u_x_*(*SIDE*_*MID*)/*W* and *ε_y_* = −δ/*H*, with *W* = *H* = 50 mm;
(3) Effective stiffness: *K* = *F_pk_*/δ, where *F_pk_* is the peak magnitude of the reaction force over the loading history;
(4) Specific energy absorption: SEA = *E_abs_*/*m*;
(5) Specific load capacity: *F_pk_*/m.

Metric (2) is a pointwise estimator that a local buckle passing through the probe can bias. It is complemented by a full-field estimator, *ν_ff_* = ū*_x_*/δ, where ū*_x_* is the area-averaged X displacement of the entire lateral free face (FRONT_FACE), the numerical analog of a digital-image-correlation average over the specimen side [33]. The definition mirrors metric (2), with *W* = *H*, so the two estimators are directly comparable. The two displacement amplitudes are, for every angle, two solutions of the same quasi-static path obtained with different increment sizes (0.05 and 0.10 mm per substep), so their lateral displacement histories must coincide at matched stroke unless the path is unstable; this reproducibility test is reported in Section 3.2. Finally, the total strain energy *E_abs_* was compared with the external work *V_ext_*; the two agree to within 0.1–0.5% for all primary configurations (Table 3), so frictional dissipation is negligible and either quantity may serve as the absorbed energy.

**Table 3:** End-of-analysis (*t* = 1 s) results for the fourteen primary configurations (intact material model, baseline mesh). *F_end_* is the reaction force at the final displacement, *F_pk_* the peak reaction force over the loading history, *E_abs_* the total strain energy and *W_ext_* = ∫F∗dδ the external work of the reaction force.

Config.	*σ_vm_*_,*max*_ (MPa)	*σ*_1,*max*_ (MPa)	*F_end_* (kN)	*F_pk_* (kN)	*E_abs_* (J)	*W_ext_* (J)
0-5	46.53	49.16	4.678	4.678	19.37	19.39
0-10	52.16	51.30	4.408	4.686	42.22	42.25
15-5	54.85	57.32	6.370	7.260	30.11	30.12
15-10	53.75	54.70	6.159	7.165	61.33	61.38
30-5	47.57	44.48	4.521	4.523	19.03	19.04
30-10	48.89	48.89	4.404	4.522	41.19	41.24
45-5	44.43	46.73	3.962	3.962	14.88	14.90
45-10	54.01	51.76	4.723	4.723	36.45	36.50
60-5	46.97	48.97	3.424	3.424	12.09	12.12
60-10	55.97	56.44	3.458	3.578	29.62	29.67
75-5	45.77	47.39	3.469	3.470	13.64	13.65
75-10	50.63	51.63	3.809	3.868	31.96	32.11
90-5	47.01	49.55	4.683	4.683	19.39	19.41
90-10	51.63	54.43	4.399	4.689	42.22	42.26

### 2.5. Damage-Bounded Energy Absorption Projection

To bound the effect of the missing fracture model without re-running the campaign, a conservative projection was applied in post-processing. Unlike the generic fracture strains that must be assumed for metals [34], PA12 possesses a directly measured ductility limit. The distinction is not merely one of convenience: damage evolution in semi-crystalline thermoplastics departs qualitatively from the behavior on which metal-derived criteria are built—the cumulative dissipated energy at failure increases rather than decreases with life—so such criteria require material-specific reformulation before they can be transferred [35]. Anchoring the bound on a measured property therefore avoids importing an assumption whose validity for this material class is not established. Concretely, PA12 possesses a directly measured ductility limit: the elongation at break of laser-sintered PA12 is 20% in the X and Y build directions (Table 1). A critical equivalent plastic strain of *ε_f_* = 0.20 was therefore adopted and is in this case a material property rather than a modeling assumption. For each configuration, the recorded time histories of the maximum equivalent plastic strain and of the total strain energy were combined to determine the fracture-onset instant *t_f_*, at which the hinge-region plastic strain first reaches *ε_f_*. The stroke reached at that instant, *δ_f_* = *δ*·*t_f_*, and the energy absorbed up to it, *E_abs_*(*t_f_*), define the damage-bounded specific energy absorption SEA_f_ = *E_abs_*(*t_f_*)/*m*. Because εf is compared against the most strained single location in the model, the projection is deliberately conservative: it flags the onset of local tearing at the critical ligament–node junction, not the exhaustion of the structure’s global capacity, and a real lattice—in which a local crack initially only redistributes load—will perform between SEA_f_ and the intact-model upper bound. Since the Z-direction elongation at break is only 10%, parts printed with the lattice plane normal to the build direction would fracture even earlier; the values reported here therefore presuppose the favorable in-plane build orientation. Because *ε_f_* is the single assumed quantity on which the projection rests, the entire calculation was repeated for *ε_f_* = 0.10, 0.15, 0.20, 0.25 and 0.30—a range that spans the full build-orientation anisotropy of PA12 and extends 50% beyond its in-plane ductility—so that the sensitivity of every conclusion to this choice could be quantified (Section 3.5).

## 3. Results

### 3.1. Global Response and Tabulated Data

Table 2 summarizes the geometric and discretization data of the seven primary lattices, Table 3 the end-of-analysis results of all fourteen configurations, Table 4 the derived metrics Table 5 reproducibility and Table 6 the damage-bounded projections. Three headline observations emerge. First, the 0° and 90° lattices are geometrically congruent (identical volume, 45,724 mm^3^) and return virtually identical responses (*F_pk_* = 4.678 and 4.683 kN at 5 mm; SEA = 456 J/kg in both cases), an internal consistency check on the parametric model and the solver settings. Second, the 15° configuration is a clear outlier in load capacity: its peak force of 7.26 kN exceeds every other lattice by at least 55% (Section 3.7 shows this peak to be an isolated spike). Third, the two solutions of each angle reproduce one another: at matched strokes of 2–5 mm the reaction forces agree to within 0.06% for six of the seven angles and to within 1.5% for the 15° lattice (Table 5). For the four architectures whose peak force is attained within the first 5 mm (0°, 15°, 30°, 90°) the two values of *F_pk_* agree to within 1.3%, whereas the 45°, 60° and 75° lattices are still hardening at 5 mm and record higher peaks in their 10 mm analyses. All forces and energies in Table 3 and Table 4 are baseline-mesh values, which Section 3.6 shows to be upper estimates.

**Table 4:** Derived performance metrics (intact material model, baseline mesh; SEA values are upper bounds). *u_x_* is the lateral displacement of the SIDE_MID probe and ū*_x_* the area-averaged lateral displacement of the free face FRONT_FACE (positive: outward; negative: inward); *v_eff_* and *v_ff_* are the corresponding single-probe and full-field effective Poisson’s ratios. The 15° entries are branch-dependent (Section 3.2).

Config.	*u_x_* Probe (mm)	ū*_x_* Face (mm)	*v_eff_* Probe (–)	*v_ff_* Full-Field (–)	*K* (N/mm)	SEA (J/kg)	*F_pk_*/*m* (kN/kg)
0-5	+4.412	+4.864	+0.88	+0.97	936	456	110.0
0-10	+7.504	+8.232	+0.75	+0.82	469	993	110.2
15-5	−0.328	−1.682	−0.07	−0.34	1452	641	154.5
15-10	−4.638	−6.709	−0.46	−0.67	717	1306	152.5
30-5	−3.792	−3.644	−0.76	−0.73	905	405	96.2
30-10	−6.026	−6.018	−0.60	−0.60	452	876	96.2
45-5	−3.177	−1.571	−0.64	−0.31	792	342	91.2
45-10	−4.385	−2.299	−0.44	−0.23	472	839	108.7
60-5	−0.631	+0.797	−0.13	+0.16	685	281	79.8
60-10	+1.910	+3.406	+0.19	+0.34	358	690	83.3
75-5	+2.657	+3.019	+0.53	+0.60	694	322	82.0
75-10	+5.814	+5.420	+0.58	+0.54	387	755	91.4
90-5	+4.472	+4.883	+0.89	+0.98	936	456	110.1
90-10	+7.597	+8.261	+0.76	+0.83	469	993	110.3

**Table 5:** Reproducibility between the two solutions of each angle (5 mm and 10 mm analyses, increment sizes 0.05 and 0.10 mm) at matched strokes of 2–5 mm: maximum absolute deviation of the single-probe and full-field lateral displacements and maximum relative deviation of the reaction force.

θ (°)	0	15	30	45	60	75	90
Max. |Δu*_x_*| probe (mm)	0.001	3.996	0.000	0.000	0.008	0.000	0.001
Max. |Δū***_x_***| full-field (mm)	0.001	2.629	0.000	0.000	0.007	0.000	0.001
Max. |Δ*F*|/*F* (%)	0.05	1.47	0.04	0.05	0.06	0.06	0.05

**Table 6:** Damage-bounded projections using the PA12 elongation at break (*ε_f_* = 0.20). *ε_pl_*_,*max*_ is the maximum equivalent plastic strain at the end of the analysis; *δ_f_* is the projected stroke at fracture onset; SEA_f_ = *E_abs_*(*t_f_*)/*m*. Entries “>5.0” indicate that the criterion is not reached within the 5 mm analysis.

Config.	*ε_pl_*_,*max*_ (–)	*δ_f_* (mm)	*E_abs_*(*t_f_*) (J)	SEA_f_ (J/kg)	SEA_f_/SEA (–)
0-5	0.140	>5.0	19.37	456	1.00
0-10	0.232	7.94	32.98	776	0.78
15-5	0.291	3.59	21.09	449	0.70
15-10	0.327	3.53	20.54	437	0.33
30-5	0.117	>5.0	19.03	405	1.00
30-10	0.219	9.40	38.55	820	0.94
45-5	0.129	>5.0	14.88	342	1.00
45-10	0.272	6.84	22.42	516	0.62
60-5	0.079	>5.0	12.09	281	1.00
60-10	0.291	7.75	21.80	508	0.74
75-5	0.129	>5.0	13.64	322	1.00
75-10	0.252	7.61	22.87	540	0.72
90-5	0.132	>5.0	19.39	456	1.00
90-10	0.222	8.46	35.38	832	0.84

### 3.2. Deformation Mechanics and Auxetic Behavior

**Figure 4:**
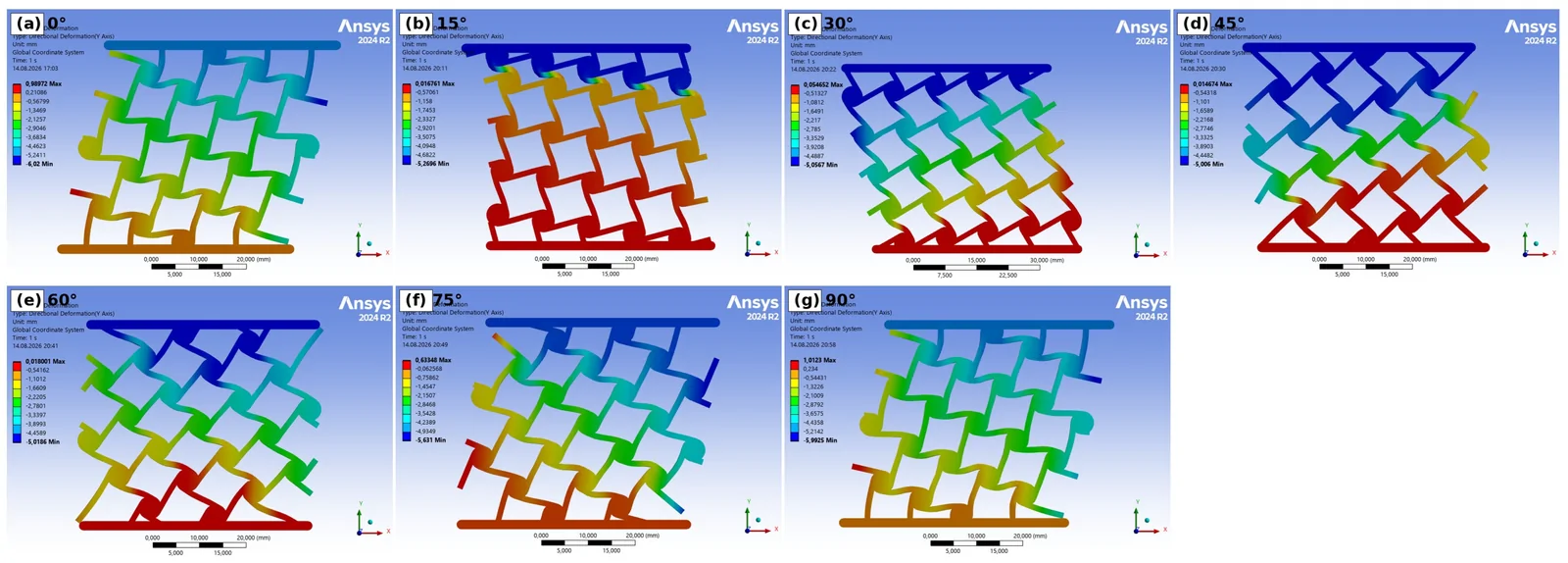
Directional (Y-axis) deformation contours at *δ *= 5 mm for *θ* = 0–90° (**a**–**g**). Note the row-wise collapse initiating at the fixed support in the auxetic configurations.

**Figure 5:**
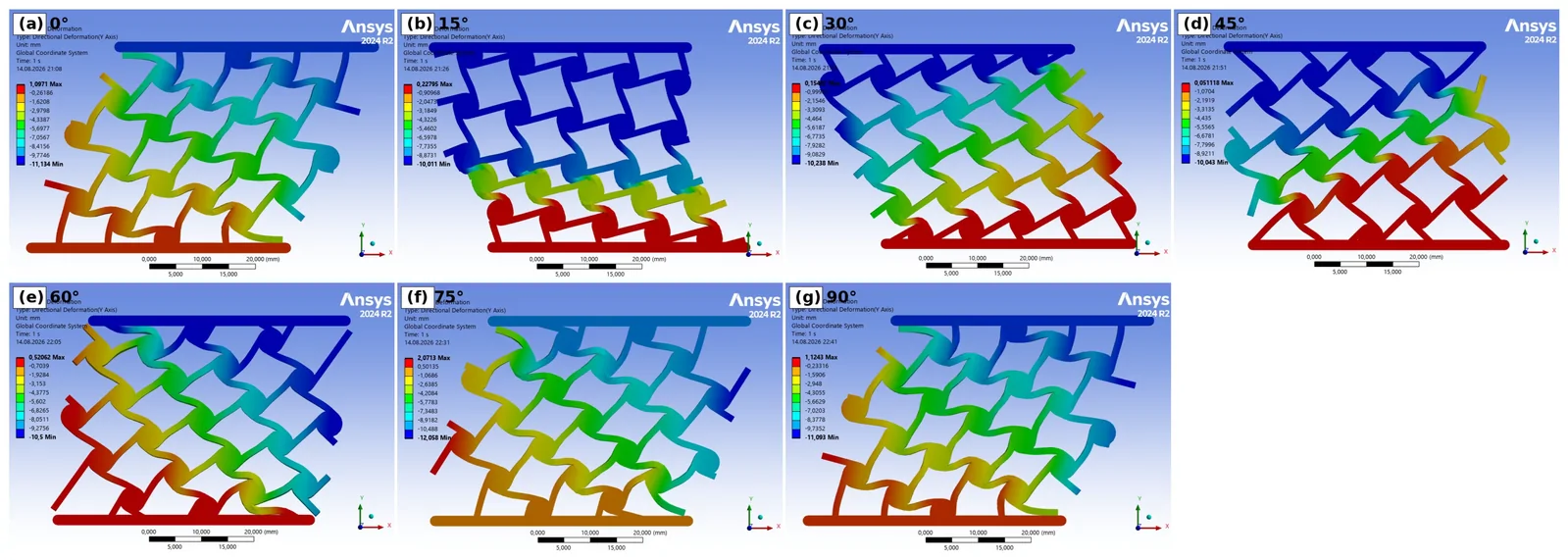
Directional (Y-axis) deformation contours at *δ* = 10 mm for *θ* = 0–90° (**a**–**g**). Densification bands and cell-wall contact are visible in the lower rows.

The Y-direction deformation fields at 5 mm and 10 mm are compared in Figure 4 and Figure 5. Two qualitatively different modes are evident. In the auxetic configurations (15–45°), compression is accommodated by co-operative rotation of the circular nodes: the ligaments wind onto the nodes, the cells fold inward, and the deformation localizes in the horizontal cell rows adjacent to the fixed support while the upper rows translate almost rigidly (Figure 4b–d and Figure 5b–d). The localization next to the support is favored by the rough lower boundary, which prevents the bottom row from relieving load by lateral sliding (Section 4.5). In the non-auxetic configurations (0°, 75°, 90°), the near-axial ligaments act as short columns; deformation spreads more uniformly and the lateral faces bulge outward in the manner of positive-ν cellular solids.

Before the Poisson data were interpreted, their reproducibility was tested with both estimators by comparing the 5 mm and 10 mm analyses at matched strokes of 2, 3, 4 and 5 mm (Table 5). Six of the seven angles reproduce to within 0.01 mm with either estimator while their reaction forces agree to within 0.06%: the two increment sizes yield the same solution and the global response is path-independent. The 15° lattice is the sole exception: its two solutions diverge by up to 4.0 mm at the probe and by 2.6 mm in the full-field average, while their reaction-force histories agree to within 1.5% at every matched stroke. The 15° architecture therefore possesses two distinct lateral collapse branches of nearly identical global stiffness, selected by the discretization rather than by a seeded lateral force (Section 3.6). No single Poisson’s ratio characterizes the 15° lattice: *v_eff_* = −0.07 (branch A) or −0.46 (branch B) at the probe, and *v_ff_* = −0.34 or −0.67 in the full-field measure.

With this qualification in place, the two estimators (Table 4) describe a well-defined auxetic window. For 0°, 30°, 75° and 90° they agree to within 0.1 at both amplitudes; for 15°, 45° and 60° they differ, and the full-field value is the one to be read: at 45° the probe overstates the auxeticity (*v_eff_* = −0.64 versus *v_ff_* = −0.31 at 5 mm; −0.44 versus −0.23 at 10 mm), and at 60° the probe registers a small inward motion at 5 mm (−0.13) whereas the face as a whole moves outward throughout the stroke (*v_ff_* = +0.16 at 5 mm and +0.34 at 10 mm), so the 60° lattice is non-auxetic at both amplitudes and the apparent sign change at the probe is a local buckle passing through SIDE_MID. The full-field ratio falls from +0.97 at 0° into negative territory at 15–45°, with a plateau of −0.60 to −0.67 across 15–30° at 10 mm (Section 3.7), weakens to −0.23 at 45°, changes sign between 45° and 50° (+0.11 at 50°) and returns to strongly positive values at 75° (+0.54) and 90° (+0.83). The auxetic window thus spans approximately 10–47°, with its strongest reproducible value at 30°. Increasing the compression from 10% to 20% strain weakens the auxetic effect at 30° (−0.73 → −0.60) and 45° (−0.31 → −0.23) as the folded ligaments align with the loading axis and the rotational lever arm shortens.

### 3.3. Stress Distribution

**Figure 6:**
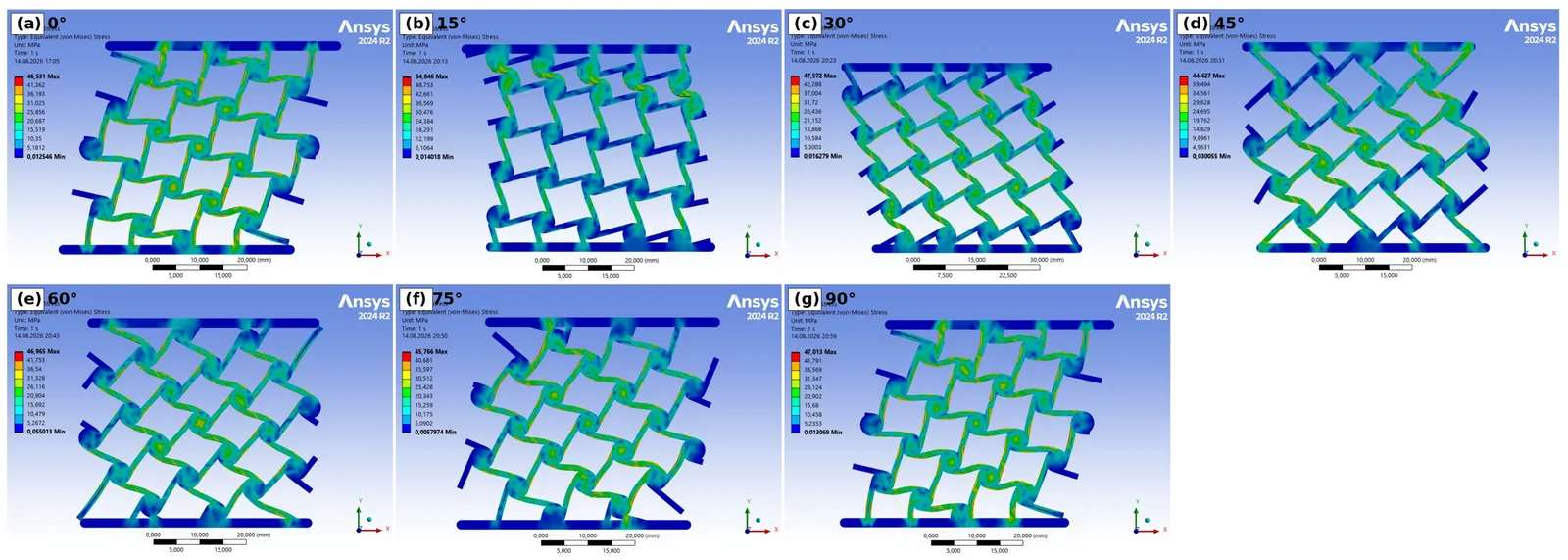
Von Mises stress contours at *δ* = 5 mm for *θ* = 0–90° (**a**–**g**). Stress concentrates at the ligament–node junctions.

**Figure 7:**
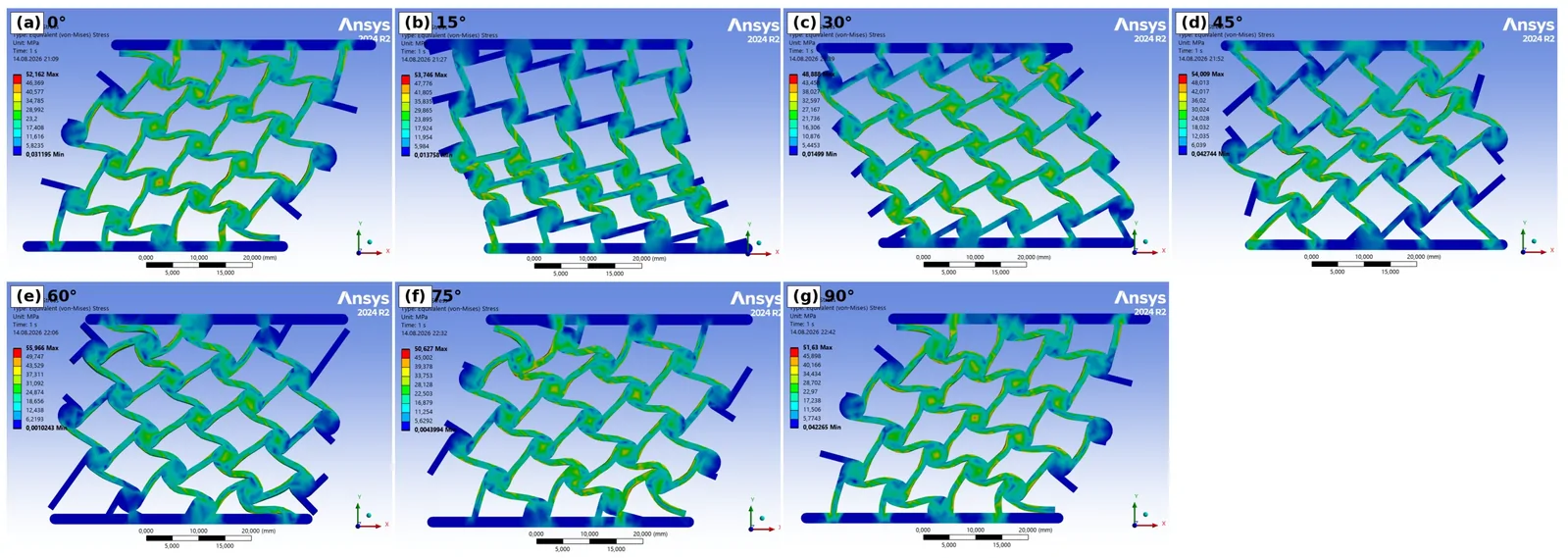
Von Mises stress contours at *δ* = 10 mm for *θ* = 0–90° (**a**–**g**).

Figure 6 and Figure 7 present the von Mises stress fields. In every configuration the highest equivalent stresses concentrate at the ligament–node junctions, where the bending moment transferred by the eccentric connection is maximal; the circular nodes themselves remain comparatively lightly stressed, acting as quasi-rigid rotational hubs. At 5 mm the maximum von Mises stress lies within a narrow band of 44.4–54.9 MPa (Table 3)—above the 35 MPa initial yield strength because of the bilinear hardening and approaching the 50 MPa ultimate strength of the parent polymer at the most heavily worked junctions. At 10 mm the maxima rise moderately to 48.9–56.0 MPa. The modest increase despite doubled strain reflects the low tangent modulus of PA12: additional deformation extends the plastic zones rather than raising local stress, which is the hallmark of an efficient plateau absorber.

Maximum principal stresses follow the same pattern, spanning 44.5–57.3 MPa, with the highest value again in the 15° lattice at 10% strain, and several configurations exceed the 50 MPa data-sheet tensile strength of PA12 locally. This comparison is not a failure criterion, since a local, multiaxial, bending-dominated stress at a notch-like junction cannot be judged against a nominal uniaxial strength and the baseline mesh over-predicts the local stress at the hinges (Section 3.6); it is a qualitative indicator that the most heavily worked junctions of the 15° lattice operate at the limit of the material’s tensile capacity. The quantitative treatment of material failure is the plastic-strain projection of Section 3.4 and its sensitivity study in Section 3.5.

### 3.4. Load-Bearing Capacity and Energy Absorption: Upper Bounds and Damage-Bounded Projections

**Figure 8:**
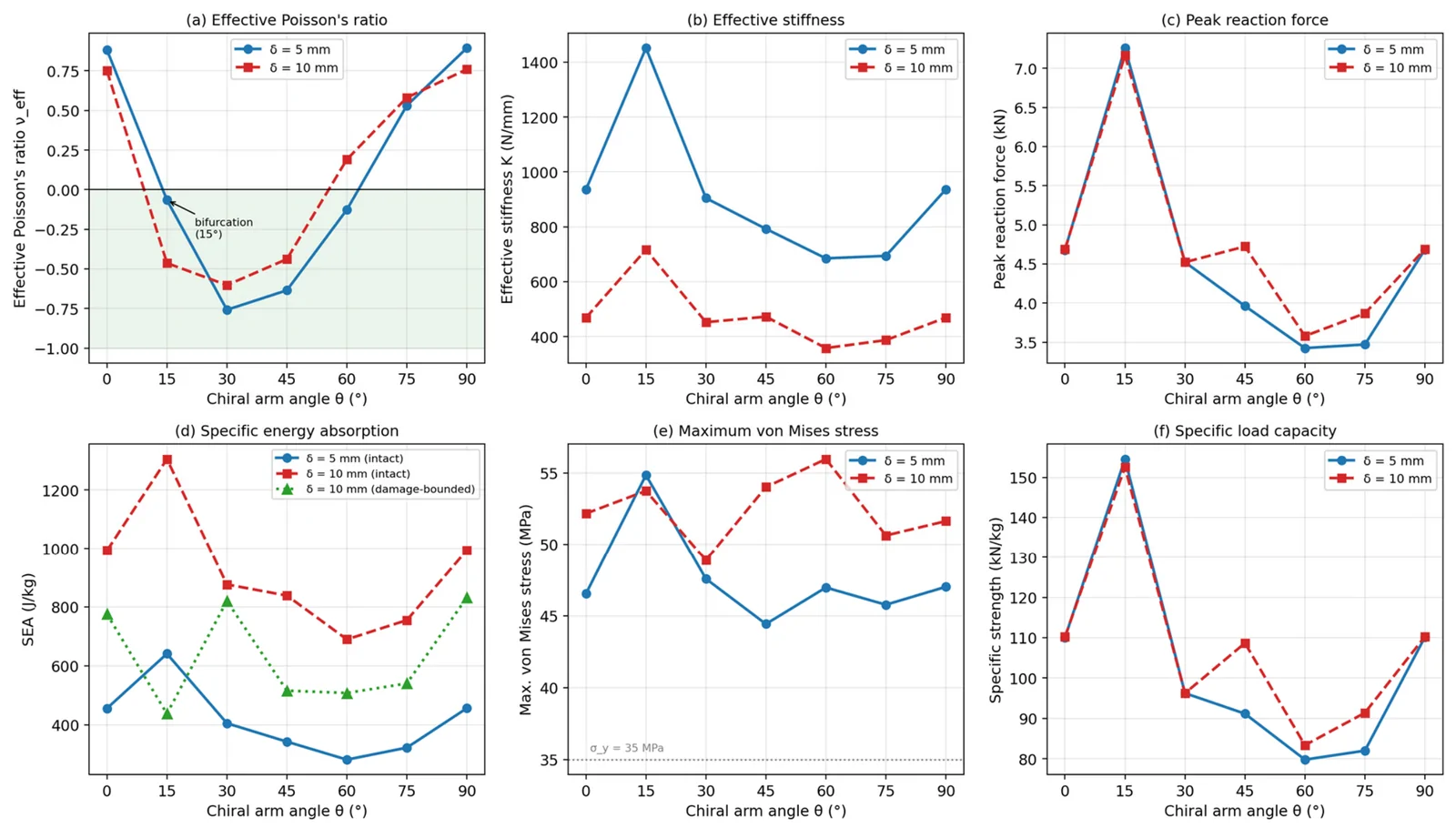
Angle dependence of the derived metrics for *δ* = 5 mm and *δ* = 10 mm:

The angle dependence of the derived metrics is mapped in Figure 8. The effective stiffness at 10% strain peaks sharply at 15° (1452 N/mm), 55% above the congruent 0°/90° limits (936 N/mm) and more than double the 60° minimum (685 N/mm). The same ranking holds for the peak force (7.26 kN at 15° versus 3.42–4.69 kN elsewhere) and survives mass normalization: the specific load capacity of the 15° lattice (154.5 kN/kg) exceeds the family average by roughly 60%, so the advantage is architectural rather than a consequence of its slightly higher relative density (0.404 versus 0.364–0.374).

Energy absorption under the intact-material assumption follows the same hierarchy. At 20% strain the 15° lattice absorbs 61.3 J (upper-bound SEA = 1306 J/kg), against 42.2 J (993 J/kg) for the 0°/90° limits and 29.6 J (690 J/kg) for the 60° minimum. For every angle, doubling the imposed displacement multiplies the absorbed energy by 2.04–2.45 while the transmitted load remains on its plateau, the signature of a plateau-type absorber. The mild super-linearity reflects the plastic hardening of the growing hinge regions and the geometric stiffening of progressively folded ligaments (the friction study of Section 3.6 rules out a contact contribution) and is most pronounced at 45° and 60° (2.45 in both cases), the two architectures whose inclined ligaments fold earliest.

The damage-bounded projections of Table 6 substantially reorder these results. At 10% strain the maximum equivalent plastic strain remains below the 0.20 ductility limit of PA12 for six of the seven angles (0.079–0.140), so those lattices operate entirely within the intact regime. The 15° lattice is the exception: it reaches *ε_f_* after 3.59 mm of stroke, i.e., at 7.2% nominal strain, and its usable SEA falls to 449 J/kg. At 20% strain every configuration crosses the limit, but at markedly different strokes: 3.53 mm for 15°, 6.84–8.46 mm for 45°, 60°, 75° and 0°, and 9.40 mm for 30°. The corresponding damage-bounded SEA values place 90° (832 J/kg), 30° (820 J/kg) and 0° (776 J/kg) at the top and 15° (437 J/kg) at the bottom, an inversion of the intact-model ranking. Two qualifications apply. The two solutions of the 15° lattice give fracture-onset strokes of 3.59 and 3.53 mm despite following different lateral branches, so the early onset is a property of the architecture rather than of the branch. And the maximum plastic strain is a local, mesh-sensitive quantity: δ*_f_* decreases with refinement (to 2.81 mm for 15° and 4.74 mm for 30° at *h* = 1.0 mm) while the ordering is preserved (Section 3.6), so the values in Table 6 are baseline-mesh upper estimates whose ranking is robust.

### 3.5. Sensitivity of the Damage-Bounded Projection to the Critical Plastic Strain

Because *ε_f_* is the single assumed quantity on which the projection rests, the calculation was repeated for the 10 mm analyses with *ε_f_* = 0.10, 0.15, 0.20, 0.25 and 0.30, a range that spans the build-orientation anisotropy of PA12 (10% elongation at break in Z, 20% in-plane) and extends 50% beyond its in-plane ductility (Table 7). The ordering of fracture onset is invariant: the 15° lattice is the first to reach the limit at every value examined (1.72, 2.23, 3.53, 5.28 and 8.46 mm, against 3.72–10.0 mm for all other angles). The energy ranking is equally robust: 15° returns the lowest SEA_f_ for every *ε_f_* up to 0.25, and only at *ε_f_* = 0.30, 50% beyond the measured value, does it overtake 30°, which retains the longest fracture-onset stroke of the auxetic configurations for every *ε_f_* ≥ 0.15. The elimination of 15° and the preference for 30° therefore do not depend on the value adopted for the fracture strain, although the absolute values of δ*_f_* and SEA_f_ do, as they also depend on mesh density (Section 3.6).

**Table 7:** Sensitivity of the damage-bounded projection (10 mm analyses, baseline mesh) to the critical equivalent plastic strain *ε_f_*: fracture-onset stroke δ*_f_* and damage-bounded specific energy absorption SEA_f_. Entries “>10” indicate that the criterion is not reached within the 10 mm stroke, in which case SEA_f_ equals the intact value.

θ (°)	δ*_f_* (mm) at *ε_f_* = 0.10	0.15	0.20	0.25	0.30	SEA_f_ (J/kg) at *ε_f_* = 0.10	0.15	0.20	0.25	0.30
0	3.72	5.36	7.94	>10	>10	316	495	776	993	993
15	1.72	2.23	3.53	5.28	8.46	185	258	437	679	1096
30	4.21	6.80	9.40	>10	>10	329	577	820	876	876
45	4.16	5.61	6.84	8.04	>10	267	398	516	634	839
60	5.56	6.73	7.75	8.92	>10	326	423	508	603	690
75	4.16	5.62	7.61	9.84	>10	253	373	540	741	755
90	3.80	5.83	8.46	>10	>10	325	548	832	993	993

### 3.6. Mesh Convergence and Sensitivity to Seeded Imperfections and Friction

**Figure 9:**
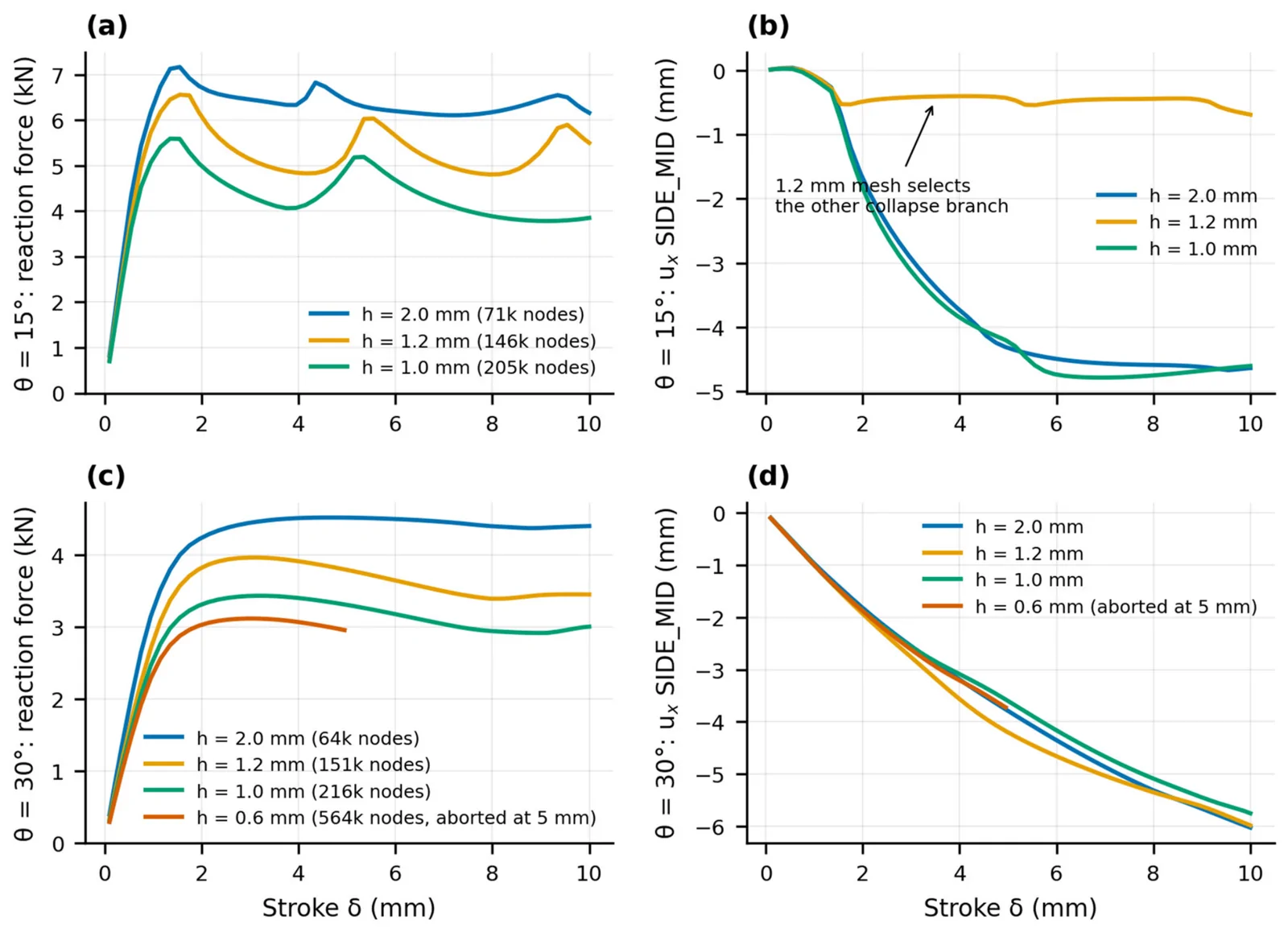
Mesh convergence: reaction force (**a**,**c**) and lateral probe displacement (**b**,**d**) versus stroke for the 15° (top) and 30° (bottom) lattices at global element sizes of 2.0, 1.2, 1.0 and 0.6 mm. Forces decrease systematically with refinement; the kinematics are converged, except that the 1.2 mm mesh of the 15° lattice selects the alternative collapse branch.

The convergence study is summarized in Table 8 and Figure 9. The finest mesh (*h* = 0.6 mm, 546,000–564,000 nodes) was not completed: the 15° solution did not converge and the 30° solution was aborted at 4.95 mm. The initial secant stiffness K*_sec_* (between 0.5 and 1.0 mm of stroke), the peak force and the absorbed energy decrease monotonically with refinement and have not converged at the baseline size: relative to *h* = 2.0 mm, the peak force is 8.5% and 22.0% lower at *h* = 1.2 and 1.0 mm for the 15° lattice, and 12.3%, 24.0% and 31.0% lower at *h* = 1.2, 1.0 and 0.6 mm for the 30° lattice, with the absorbed energy following (−16% to −31%). The sequence is not yet asymptotic, so no Richardson extrapolation is attempted; the finest level suggests a converged plateau force roughly 30–35% below the baseline value. Since the through-thickness discretization is the same at every level, the refinement acts along the ligaments, and the plastic-strain column shows what it resolves: the hinges at the ligament–node junctions localize with refinement (ε_pl,max_ rising from 0.22 to 0.47 at 30° and from 0.33 to 0.50 at 15°), whereas the 2.0 mm mesh, in which a hinge spans a single element along the ligament, is artificially stiff.

**Table 8:** Mesh convergence of the 15–10 mm and 30–10 mm configurations. Percentages are changes relative to the baseline mesh (*h* = 2.0 mm); *K_sec_* is the secant stiffness between 0.5 and 1.0 mm of stroke; *u_x_* (4.9 mm) is the probe displacement at the last stroke reached by all meshes. The *h* = 0.6 mm solution of the 15° lattice did not converge and is not listed; that of the 30° lattice was aborted at 4.95 mm. n/a: not available; the *h* = 0.6 mm solution of the 30° lattice was aborted by the solver at 4.95 mm of stroke, so the quantities defined at 10 mm (*E*abs and *u*x) could not be evaluated for this mesh.

*θ* (°)	*h* (mm)	Nodes	*K_sec_* (N/mm)	*F_pk_* (N)	*E_abs_* (10 mm) (J)	*u_x_* (4.9 mm) (mm)	*u_x_* (10 mm) (mm)	*ε_pl_*_,*max*_	δ*_f_* (mm)
15	2.0	70,915	4663	7166	61.3	−4.29	−4.64	0.327	3.53
15	1.2	146,099	4584 (−1.7%)	6557 (−8.5%)	51.6 (−15.8%)	−0.42	−0.69	0.353	3.12
15	1.0	205,489	3670 (−21.3%)	5591 (−22.0%)	42.6 (−30.5%)	−4.19	−4.60	0.497	2.81
30	2.0	64,224	2780	4522	41.2	−3.73	−6.03	0.219	9.40
30	1.2	151,170	2482 (−10.7%)	3966 (−12.3%)	34.3 (−16.8%)	−4.15	−5.98	0.366	5.44
30	1.0	215,575	2198 (−20.9%)	3435 (−24.0%)	29.8 (−27.7%)	−3.55	−5.75	0.465	4.74
30	0.6	563,903	2012 (−27.6%)	3119 (−31.0%)	n/a	−3.70	n/a	0.255	3.87

Two properties of the solution are converged or nearly so. First, the lateral kinematics: the *u*_x_ histories of the 30° lattice at the four mesh levels lie within 0.5 mm of one another over the whole stroke and within 0.3 mm at 10 mm (−6.03, −5.98 and −5.75 mm), and the 15° lattice returns the same branch-B history at *h* = 2.0 and 1.0 mm (−4.64 and −4.60 mm at 10 mm) while the 1.2 mm mesh follows branch A (−0.69 mm), a further manifestation of the bifurcation of Section 3.2 rather than a lack of convergence. Second, the relative ordering of the architectures: across the three complete mesh levels the 15°/30° ratio stays within 1.67–1.85 for the initial stiffness, 1.58–1.65 for the peak force and 1.43–1.51 for the absorbed energy, and the 15° lattice reaches the ductility limit before the 30° lattice at every level (δ*_f_* = 3.53/9.40, 3.12/5.44 and 2.81/4.74 mm). The discretization error is systematic and of similar magnitude for both architectures, so it shifts the family as a whole. All absolute forces, stiffnesses, energies and fracture-onset strokes reported at the baseline mesh are therefore upper estimates carrying a discretization uncertainty of about 20–30%, whereas the Poisson’s ratios, the auxetic window, the bifurcation and the ranking of the architectures are not affected. Where absolute figures are quoted in the following sections they are therefore given together with their value on the finest complete mesh (*h* = 1.0 mm, Table 8): for the 30° lattice a peak force of 4.52 (3.44) kN, an absorbed energy of 41.2 (29.8) J and a fracture-onset stroke of 9.40 (4.74) mm; for the 15° lattice 7.17 (5.59) kN, 61.3 (42.6) J and 3.53 (2.81) mm.

**Figure 10:**
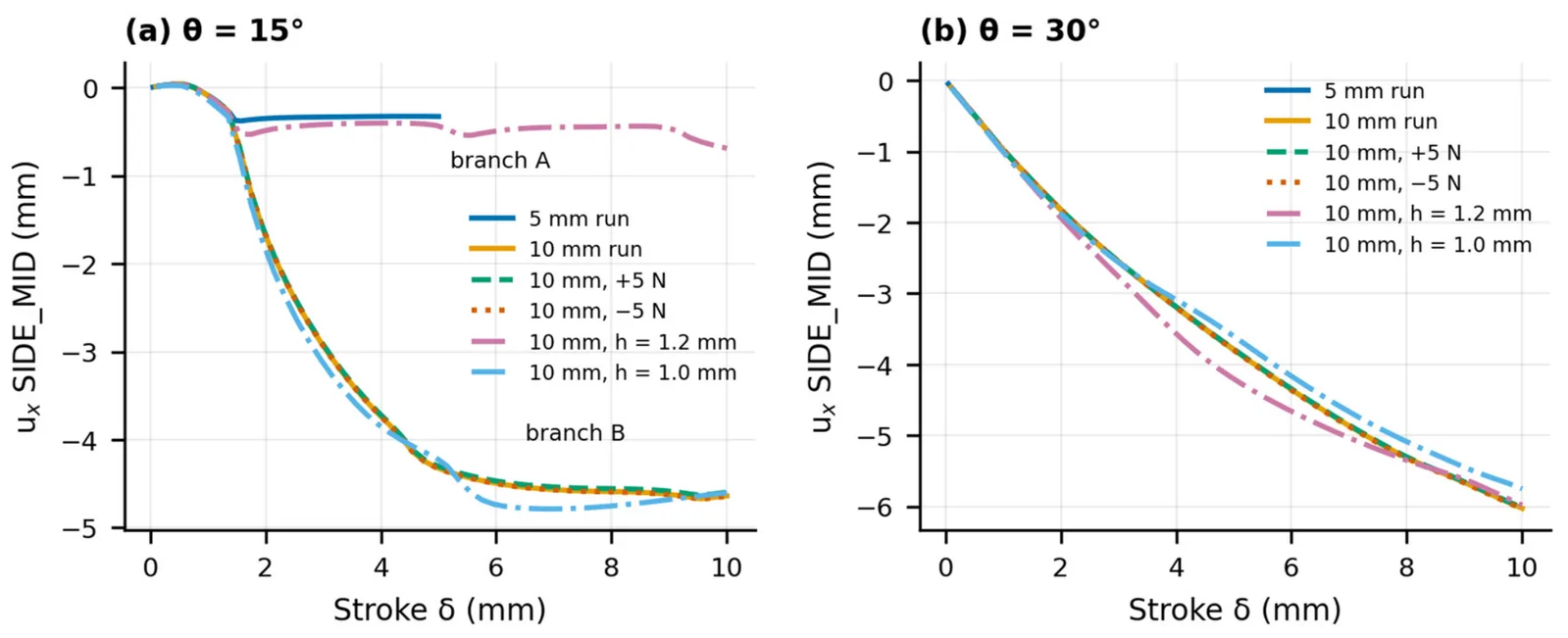
Lateral probe displacement versus stroke for the 5 mm and 10 mm solutions, the two seeded-imperfection solutions (±5 N) and the refined-mesh solutions: (**a**) the 15° lattice follows one of two branches, A (5 mm run, 1.2 mm mesh) or B (10 mm runs, 1.0 mm mesh); (**b**) the 30° lattice follows a single path.

The seeded-imperfection analyses (±5 N in X at mid-height, Figure 10) separate the two architectures sharply. The 30° lattice is insensitive to every perturbation: the seeds change the lateral displacement at 10 mm by less than 0.01 mm and the peak force by less than 0.1%, and its collapse path is unique across increment sizes and mesh levels. The seeds do not switch the branch of the 15° lattice: both perturbed solutions follow branch B (*u_x_* = −4.60 and −4.64 mm at 10 mm against −4.64 mm unperturbed), so a lateral force of 5 N, 0.07% of the peak load, is not the perturbation that selects the branch. The branch is instead selected by the discretization: on the baseline mesh the 0.05 mm increment yields branch A and the 0.10 mm increment branch B, and the 1.2 mm mesh yields branch A while the 1.0 mm mesh yields branch B. The branches separate at about 1.5 mm of stroke, immediately after the peak force, where the force–displacement curve exhibits a limit point and the lattice can proceed along either of two post-peak paths of almost identical energy; the energy separation between them must therefore be smaller than the numerical perturbations of the model. Which branch a physical specimen would follow, and at what imperfection amplitude the choice becomes deterministic, cannot be established from the present analyses (Section 4.6).

The friction coefficient has no measurable influence. For *μ* = 0.1, 0.2 and 0.3 every reported quantity of the 15° and 30° lattices (peak and end force, absorbed energy, lateral displacement, maximum plastic strain and maximum von Mises stress) is identical to five significant figures: within the 10 mm stroke the self-contact pairs either do not close or remain in the sticking regime, in which case the tangential force is independent of *μ*. Consistently, the three 5 mm models for which the contact search generated no contact pairs (30°, 45°, 75°) coincide with their contact-equipped 10 mm counterparts to within 0.06% in force over the common stroke (Table 5), and the strain energy equals the external work to within 0.5% (Table 3). Within 20% nominal strain the response is therefore governed by ligament bending, plastic hinge formation and the geometric reorientation of the folded ligaments, not by contact, and the uncertainty in *μ* for printed PA12 surfaces does not propagate into any conclusion of this paper.

### 3.7. Refined Angular Sweep and Density-Normalized Comparison

**Figure 11:**
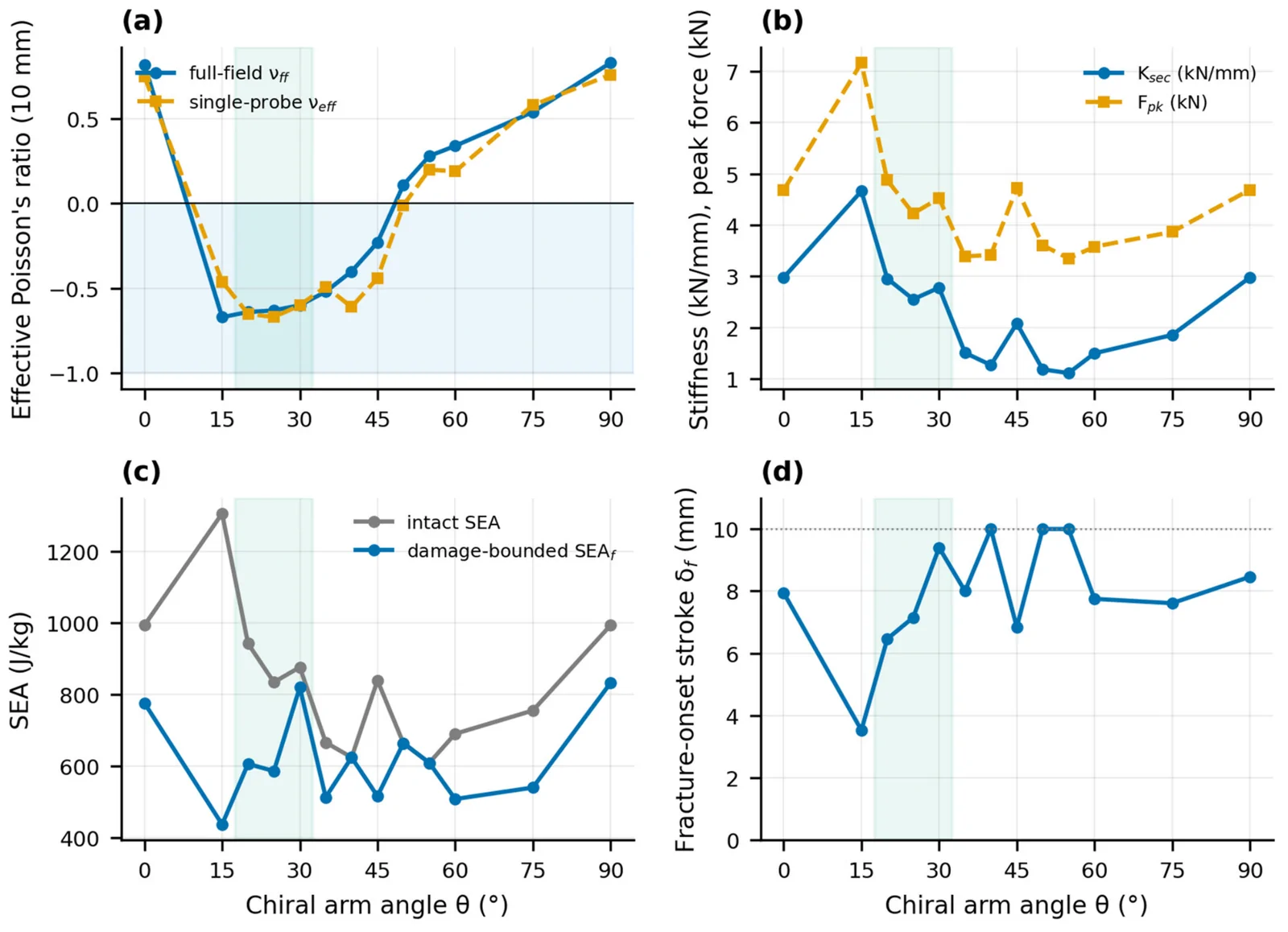
Angular map at 5° resolution (10 mm analyses): (**a**) full-field and single-probe effective Poisson’s ratios (shaded: auxetic); (**b**) initial secant stiffness and peak force; (**c**) intact and damage-bounded specific energy absorption; (**d**) fracture-onset stroke (ε*_f_* = 0.20; 10 mm = not reached). The green band marks the 20–30° auxetic plateau.

Six additional lattices (20°, 25°, 35°, 40°, 50° and 55°) were analyzed at 10 mm on the baseline mesh, giving a thirteen-point map at 5° resolution across 15–60° (Table 9, Figure 11). (i) The 15° peak in stiffness and load capacity is an isolated spike: the initial secant stiffness drops from 4663 N/mm at 15° to 2956 N/mm at 20° and 2553 N/mm at 25° before recovering slightly to 2780 N/mm at 30°, and the peak force falls from 7.17 kN to 4.88 kN between 15° and 20°. (ii) The auxetic magnitude forms a plateau rather than a peak: ν_ff_ = −0.67, −0.64, −0.63 and −0.60 at 15°, 20°, 25° and 30°, then −0.52 (35°), −0.40 (40°) and −0.23 (45°). (iii) The transition to positive Poisson’s ratio lies between 45° and 50° (−0.23 → +0.11), with 55° and 60° at +0.28 and +0.34. (iv) Within the plateau the damage-bounded metrics single out 30°: its fracture-onset stroke of 9.40 mm and SEA_f_ of 820 J/kg exceed those of 20° (6.46 mm, 606 J/kg), 25° (7.15 mm, 586 J/kg) and 35° (8.00 mm, 513 J/kg). The 40–55° lattices do not reach the ductility limit within 10 mm but offer weak or no auxeticity. The 30° optimum is therefore confirmed at 5° resolution: 20–30° is a design plateau in Poisson’s ratio within which 30° is the most damage-tolerant member and the one furthest from the 15° instability.

Because the relative density varies with the number of complete cells in the envelope (Section 2.1), stiffness and peak force were also normalized by the Gibson–Ashby scaling laws for bending-dominated cellular solids [36], ${\rho_{\mathrm{rel}}}^{2}$ and ${\rho_{\mathrm{rel}}}^{1.5}$ respectively (last two columns of Table 9). The normalization changes no ranking, and the 15° and 30° lattices, which have identical relative density (0.404) and cell count (20), still differ by 60% in stiffness and peak force and by a factor of two in damage-bounded stroke; the contrast between them is purely architectural.

**Table 9:** Refined angular sweep at 10 mm stroke (baseline mesh, intact material model) merged with the seven primary angles, with density-normalized stiffness and peak force. *K_sec_* is the secant stiffness between 0.5 and 1.0 mm of stroke; δ*_f_* and SEA_f_ use ε*_f_* = 0.20.

*θ* (°)	${\rho_{\mathrm{rel}}}^{2}$	*K_sec_* (N/mm)	*F_pk_* (N)	SEA (J/kg)	ν*_ff_* (–)	*ε_pl_*_,*max*_	δ*_f_* (mm)	SEA_f_ (J/kg)	$K_{\sec}/{\rho_{\mathrm{rel}}}^{2}$	$F_{\mathrm{pk}}/{\rho_{\mathrm{rel}}}^{1.5}$
0	0.366	2979	4686	993	+0.82	0.232	7.94	776	22,264	21,182
15	0.404	4663	7166	1306	−0.67	0.327	3.53	437	28,560	27,899
20	0.390	2956	4875	942	−0.64	0.217	6.46	606	19,435	20,019
25	0.390	2553	4227	834	−0.63	0.210	7.15	586	16,830	17,389
30	0.404	2780	4522	876	−0.60	0.219	9.40	820	17,011	17,593
35	0.355	1509	3388	666	−0.52	0.226	8.00	513	11,969	16,017
40	0.353	1270	3421	624	−0.40	0.178	>10	624	10,174	16,294
45	0.374	2082	4723	839	−0.23	0.272	6.84	516	14,894	20,658
50	0.351	1186	3608	664	+0.11	0.183	>10	664	9653	17,387
55	0.349	1113	3349	609	+0.28	0.193	>10	609	9140	16,252
60	0.369	1499	3578	690	+0.34	0.291	7.75	508	10,990	15,942
75	0.364	1857	3868	755	+0.54	0.252	7.61	540	14,004	17,602
90	0.366	2979	4689	993	+0.83	0.222	8.46	832	22,262	21,193

## 4. Discussion

### 4.1. Why the Arm Angle Governs the Mechanism: Moment Arm and Mode Competition

The results can be rationalized through the moment arm that couples the axial load path to node rotation. In a tetra-chiral cell, the force transmitted along a ligament acts at an offset from the node center; the offset—and hence the torque per unit load—grows as the ligament orientation approaches tangency to the node circle. At *θ* = 0° and 90° the ligaments meet the nodes nearly radially: the moment arm vanishes, node rotation is not activated, and the vertical struts carry load in axial compression. The response is stretch-dominated, comparatively stiff, and laterally expansive (*ν_eff_* = +0.88 to +0.89), essentially that of a conventional grid with circular joints. The congruence of the 0° and 90° responses—identical to within 0.1% in force and energy—follows directly from the 90° rotational symmetry of the tetra-chiral node connectivity and provides a strong internal validation of the numerical campaign.

At *θ* = 15° the geometry approaches the canonical chiral condition in which ligaments are nearly tangent to the nodes. The ligaments retain a large axial component along the loading direction, preserving a stiff load path (hence the *K* and *F_pk_* maxima), while the tangential offset begins to convert compression into node rotation. This intermediate state is precisely why the configuration is unstable. At 15° the two load-transfer mechanisms available to a chiral cell, direct axial compression of the near-vertical ligaments and torque-driven rotation of the nodes, are of comparable energetic cost, so neither can establish itself as the governing kinematics. The lattice is, in effect, unable to decide how to collapse, and the choice is made by an asymmetry smaller than a 5 N lateral force, supplied by the discretization itself (Section 3.6). This is the mechanical origin of the bifurcation: both branches cost nearly the same energy, so all solutions reproduce the global force almost exactly, while the branches are geometrically distinct, so the lateral kinematics differ by an order of magnitude. Mode competition of this kind has been identified as a governing higher-order effect in the crashworthiness of thin-walled absorbers, where it likewise controls performance ranking [37]. The refined sweep shows how narrow the competing regime is: at 20° the rotational path is already dominant, stiffness has fallen by 37% and the response is single-branched. As *θ* increases to 30°, bending progressively replaces stretching as the primary transfer mode, stiffness falls from 1452 to 905 N/mm, and the lattice becomes both strong and reproducibly auxetic. Beyond ≈45° the sense of the ligament eccentricity relative to the compression axis reverses the rotation demand; the cells behave increasingly like an inclined-strut frame that sways laterally outward, and the full-field Poisson’s ratio changes sign between 45° and 50°. The 50° lattice, with ν*_ff_* = +0.11 at 20% strain, sits closest to this mechanistic boundary.

### 4.2. Effect of Deformation Amplitude

The comparison between the 5 mm and 10 mm analyses delineates two regimes. Up to 10% strain the structures have already yielded (the reaction force reaches its plateau within the first 1–2 mm of stroke) but geometric reconfiguration is moderate and, for six of seven angles, plastic strains remain below the ductility limit of PA12. Between 10% and 20% strain, geometric non-linearity dominates: node rotations become finite and ligaments fold onto the nodes (Figure 5). Three quantitative signatures identify this transition. First, force saturation: for the architectures that reach their peak within 5 mm the two amplitudes return *F_pk_* within 1.3%, and the force histories of all seven angles coincide at matched stroke, so the additional stroke is traversed on the plateau. Second, super-linear energy scaling (2.16–2.45 for a doubled displacement), which the friction study of Section 3.6 attributes to plastic hardening in the growing hinge regions and to the geometric stiffening of progressively folded ligaments rather than to contact. Third, the weakening of the auxetic effect at 30° and 45° between the two amplitudes, which by the same argument reflects the shortening of the rotational lever arm as the folded ligaments align with the loading axis.

For a polymer this second regime carries a penalty absent in a metallic lattice. PA12 tolerates only 20% plastic strain before separation, whereas the hinge regions accumulate 0.22–0.33 by the end of the 10 mm analyses. Consequently, the deep-compression regime that generates the largest absolute energies is precisely the regime in which the intact-material assumption becomes least tenable—which is why the damage bound reorders the ranking so decisively.

### 4.3. Why the 15° Architecture Is Rejected: Three Independent Criteria

The intact-material ranking rewards architectures that funnel load through few, highly worked plastic hinges—exactly the mechanism that exhausts local ductility first. It is important to establish that the resulting rejection of the 15° architecture does not rest on the damage projection alone. Three criteria, mutually independent in the sense that each remains valid if the other two are discarded, converge on the same conclusion.

1. Mode instability: The bifurcation of Section 3.2 and Section 3.6 is a purely kinematic finding. It requires no damage model or ductility assumption: solutions of the same path obtained with different increment sizes and mesh densities yield lateral displacements differing by 4 mm at forces within 1.5%, and a seeded lateral force of ±5 N does not remove the ambiguity. A structure whose collapse direction is not uniquely determined cannot be specified for an application in which the lateral response is the functional requirement, which is precisely the case for an auxetic component. On this ground alone the 15° lattice is unsuitable, and no refinement of the damage treatment can rehabilitate it.
2. Stress-based indication: The 15° lattice develops the highest maximum principal stress of the family, 57.3 MPa, above the 50 MPa data-sheet tensile strength of PA12, already at 10% strain. This is an indicator rather than a failure criterion (Section 3.3), but it points in the same direction as the other two criteria and is independent of the plastic-strain projection.
3. Damage-bounded energy: The projection, which indicates the onset of local tearing at the most strained hinge rather than structural failure, places that onset at 3.5–3.6 mm of stroke for the 15° lattice, consistently from both solutions despite their different branches; the 15° lattice reaches the ductility limit before the 30° lattice at every mesh level (Section 3.6) and for every fracture strain from 0.10 to 0.30 (Section 3.5), that is, for any ductility PA12 could plausibly possess.

Taken together, these make the 15° architecture the least suitable choice for a printed energy absorber despite its superior intact-model figures. Conversely, the 30° architecture spreads plasticity over many hinges, postponing fracture onset to 9.40 mm, the longest usable stroke of the auxetic window, while delivering 820 J/kg of damage-bounded SEA and retaining the plateau value of auxeticity at both amplitudes; the refined sweep confirms it as the most damage-tolerant member of the 20–30° plateau and the one furthest from the 15° instability. It is therefore identified as the balanced optimum for polymeric applications, its absolute figures being baseline-mesh upper estimates (Section 3.6).

Two secondary conclusions follow. The non-auxetic 0°/90° limits achieve the highest damage-bounded SEA (776–832 J/kg) but offer none of the functional benefits of a negative Poisson’s ratio; they are the appropriate choice only where pure energy absorption is required. And the 15° upper-bound performance is not irrecoverable: local reinforcement of the ligament–node junctions by filet enlargement or graded wall thickness is an established remedy for this failure mode [26] and would be the natural route to exploiting its exceptional specific load capacity of 154.5 kN/kg. Definitive adjudication requires an explicit damage model with element deletion [34], designated as the immediate next step.

### 4.4. Application Domains

Within its quasi-static scope, the angle sweep provides a baseline screening map for printable PA12 components, a map of the architecture’s intrinsic structural mechanics onto which rate and inertial effects must later be superimposed rather than a ready design chart for impact-rated parts. Where stable auxeticity under deep compression is the functional requirement (conformal padding that densifies beneath an indenter, synclastic sandwich cores for curved panels [12,23,38], or expandable devices exploiting radial–axial coupling) the 30° architecture is recommended, with 20–25° as alternatives of equal auxeticity and somewhat lower damage tolerance. Where maximum energy dissipation per unit mass is the sole criterion and lateral behavior is immaterial, the 0°/90° limits are preferable. The 50° configuration, with a near-zero full-field Poisson’s ratio at 20% strain, offers a laterally neutral lattice useful where reaction against enclosure walls must be minimized. As the synthesis of multifunctional lattice design by He et al. [39] emphasizes, energy-absorption metrics acquire engineering meaning only against the competing requirements of a component (mass, stiffness, lateral behavior, manufacturability), and the map is offered in that spirit. All members of the family are geometrically printable in PA12 at the 1.2 mm wall thickness by either laser sintering or MJF [24,25]. It must be underlined that these indications are quasi-static. PA12 is viscoelastic and markedly rate-sensitive, and chiral lattices exhibit measurable dynamic enhancement of both plateau stress and collapse-band ordering [33,40]; consequently, the present maps quantify the architectural contribution to performance with rate and inertial contributions deliberately removed. This separation is the value of a baseline study: it isolates a variable that a coupled dynamic analysis cannot cleanly resolve, and it supplies the reference against which any subsequent rate-dependent model can be verified. Any statement about impact-rated hardware, however, must await the dynamic analyses and experiments outlined in Section 4.5 and Section 4.6.

### 4.5. Verification Status and Comparison with Published Experiments

No companion experiments were performed on the investigated geometries, and the model is not claimed to be validated. Its verification status is as follows: the congruent 0° and 90° lattices reproduce each other to within 0.1%; solutions with different increment sizes reproduce force to within 0.06% and lateral displacement to within 0.01 mm for six of seven angles (Table 5), the exception being the 15° bifurcation; strain energy equals external work to within 0.5%; the response is independent of the friction coefficient; and the kinematics, the auxetic window and the ranking are mesh-independent, whereas absolute forces, energies and fracture-onset strokes are upper estimates with a discretization uncertainty of about 20–30% (Section 3.6). Two aspects of the model were not varied and deserve explicit discussion. First, the boundary conditions—the fully fixed lower plate is a rough-platen idealization that anchors the first collapse band to the bottom row, whereas physical tests use lubricated or dry steel platens on which the specimen ends can slide. A frictionless or frictional-sliding lower boundary would relax the constraint on the bottom row, lower the plateau force slightly, and could move the first collapse band to an interior row or distribute it over several rows, as observed experimentally for tetra-chiral PA12 by Alomarah et al. [25]; the effective Poisson’s ratio, which depends on the kinematics of the free face away from the platens, is expected to be affected only secondarily, but the 15° branch selection, which occurs at the limit point immediately after the peak force, could be sensitive to the platen constraint. Second, imperfections—the seeded ±5 N lateral force did not select the branch of the 15° lattice, whereas printed specimens carry geometric imperfections that are far larger perturbations. A ligament-thickness deviation of 0.1 mm, typical of powder-bed-fusion PA12, changes the bending stiffness of a 1.2 mm ligament by about 25%, and node-position or platen-alignment errors introduce load eccentricities orders of magnitude larger than 5 N; in a physical 15° specimen the branch would therefore almost certainly be selected deterministically by its particular imperfection pattern, but in a direction that cannot be predicted from the nominal design, and that would differ from specimen to specimen. The bifurcation is thus expected to appear experimentally as scatter in the lateral response of nominally identical 15° specimens, which is the observable that the proposed experiments should target. The comparison with published experiments below is a consistency check on the order of magnitude of the stiffness, made on lattices of different geometry, relative density and manufacturing route; it is not a validation of the present model.

Externally, the closest available benchmark is the study of Alomarah et al. [25], who compression-tested MJF PA12 tetra-chiral honeycombs and reported a measured structural modulus of 20–22 MPa (FE prediction 21 MPa) at a relative density of 0.24. The present lattices, at relative densities of 0.364–0.404, exhibit initial moduli of 31.5–165 MPa on the baseline mesh. Normalizing both by their respective parent moduli and applying the Gibson–Ashby relation for cellular solids, *E*/*E*_{*s*} = *C*(*ρ*_{*rel*})^{*n*} [36], the pair (0.24, 21/1300) and the present 30° result imply an exponent *n* ≈ 1.9 on the baseline mesh and *n* ≈ 1.5 with the finest-mesh stiffness; the 0°/90° result implies *n* ≈ 2.4. These values lie between the stretch-dominated (*n* = 1) and bending-dominated (n* = 2) limits, so the computed stiffnesses are of the magnitude that independent experimental data on a PA12 tetra-chiral lattice lead one to expect. Two further qualitative agreements support the model: the layer-by-layer collapse localized in a single row, described experimentally in [25] for the tetra-chiral specimen, is reproduced in Figure 4 and Figure 5; and the friction coefficient adopted here (*μ* = 0.2) is close to the *μ* = 0.25 measured for MJF PA12 in that work, although the present results do not depend on it (Section 3.6).

One caveat is retained: penalty-based enforcement of self-contact can artificially stiffen deeply densified states, so force levels at strokes beyond the 20% strain analyzed here would carry a numerical-stiffening uncertainty; within the present stroke the contact pairs are inactive or sticking (Section 3.6). The designated experimental program comprises laser powder bed fusion of 15°, 20° and 30° prototypes in the in-plane build orientation; repeated quasi-static compression tests (at least three specimens per geometry) following ISO 13314 with DIC-based full-field lateral strain measurement, directly comparable with the FRONT_FACE estimator; tensile characterization of the same powder batch to replace the data-sheet card; and calibration of mesh level, lower-platen boundary condition and imperfection amplitude on force–displacement response, deformation mode and lateral strain.

### 4.6. Limitations and Present Study

The conclusions of this work are bound by the following limitations, which are stated here in one place so that the scope of each claim can be judged.

Purely numerical evidence. No experiments were performed on the investigated geometries. The credibility of the model rests on the verification program of Section 3.2, Section 3.5, Section 3.6 and Section 3.7 (internal consistency, reproducibility between solutions, mesh, imperfection and friction sensitivity) and on an order-of-magnitude comparison with published measurements on a lattice of different geometry, relative density and manufacturing route (Section 4.5). The model is therefore verified but not validated, and none of its quantitative predictions have been confirmed against a physical specimen.

Unconverged forces and energies. The reaction force, the initial stiffness, the absorbed energy and the fracture-onset stroke are not converged at the baseline mesh (h = 2.0 mm). They decrease monotonically as the mesh is refined, by 22–24% in peak force and 28–31% in absorbed energy at h = 1.0 mm, and the decrement between successive levels had not yet entered the asymptotic range, so no converged value can be extrapolated. The finest mesh attempted (h = 0.6 mm, about 560,000 nodes) did not reach completion: the 15° solution did not converge, and the 30° solution was aborted by the solver at 4.95 mm of stroke, so the finest level supplies only a partial reference for one configuration. All absolute forces, stiffnesses, energies and damage-bounded strokes reported in this paper are consequently upper estimates whose true values are expected to lie 20–35% lower, and they should not be used as design allowables. What the convergence study does establish is that the lateral kinematics, the effective Poisson’s ratios, the auxetic window and its transition, and the ranking of the architectures are unchanged across all mesh levels solved; the comparative conclusions are the ones the study supports.

Undecided collapse branch of the 15° lattice. Solutions of the same 15° geometry follow one of two lateral collapse branches separated by about 4 mm of lateral displacement at essentially identical load, and the branch is selected by the discretization (increment size and mesh density) rather than by a seeded lateral force of ±5 N. The energy difference between the branches is therefore smaller than the numerical resolution of the model, and the present analysis cannot determine which branch is stable, which a physical specimen would follow, or the imperfection amplitude at which the choice becomes deterministic. The paper accordingly makes no claim about the direction of the lateral response of the 15° lattice; its conclusion is limited to the statement that this response is not uniquely determined by the nominal design. Material description and damage: the bilinear card is constructed from data-sheet values rather than calibrated against a measured true stress–plastic strain curve (Section 2.2), and the analysis carries no active damage model, so the fracture-limited response is bracketed rather than resolved. The limitation is shared by the PA12 chiral-lattice models of [25] and is mitigated by reporting intact and damage-bounded bounds anchored on a measured property (Section 3.5); post-initiation load redistribution, which places the true response between the bounds, requires a calibrated ductile damage model with element deletion, for which formulations developed for semi-crystalline thermoplastics offer a basis [35]. Boundary conditions and imperfections. The lower face plate is fully fixed (a rough-platen idealization) and the upper plate is frictionless; neither a lubricated nor a frictional-sliding lower platen was analyzed, and the position of the first collapse band next to the support is likely to depend on this choice (Section 4.5). Geometric imperfections representative of printed ligaments (thickness and node-position deviations), which are far larger perturbations than the ±5 N load seed, were not modeled.

Scope of the constitutive and manufacturing assumptions. The analyses are quasi-static and the material model is rate-independent, so the viscoelasticity and strain-rate sensitivity of PA12 are not represented; the isotropic card describes only the favorable in-plane build orientation of an anisotropic printed material; geometric printability at 1.2 mm does not guarantee equal dimensional accuracy or equal local properties at every arm angle; and the effects of moisture uptake and powder aging are not included.

Metrics. The full-field lateral estimator averages the displacement of one lateral face; the two lateral faces of a chiral lattice are not mirror images, and the single-probe estimator was shown to be unrepresentative for three of the seven angles. The energy metrics use the total strain energy, which equals the external work to within 0.5% only because frictional dissipation is negligible in the present model.

### 4.7. Outlook

The order in which these limitations should be removed follows from their weight. The first step is a mesh-refined re-analysis of the family at h ≤ 1.0 mm with hinge-focused refinement and a length-scale-regularized damage description, so that both the forces and the hinge strain converge. The second is the experimental program of Section 4.5: printed 15°, 20° and 30° specimens tested repeatedly under ISO 13314 with DIC-based lateral strain measurement, together with tensile characterization of the same powder batch; for the 15° lattice the observable of interest is the specimen-to-specimen scatter of the lateral response, which is the experimental signature of the undecided branch. Boundary-condition and geometric-imperfection sensitivity studies, an explicit-dynamics campaign on the 15° and 30° lattices, and a calibrated ductile damage model with element deletion complete the pathway from the present baseline towards validated, impact-rated design.

## 5. Conclusions

A parametric, geometrically and materially non-linear finite element study of additively manufacturable PA12 tetra-chiral lattices was performed under quasi-static compression, sweeping the chiral arm angle from 0° to 90° in 15° steps (5° across the auxetic window) within a fixed 50 × 50 × 50 mm envelope at displacement amplitudes of 5 mm and 10 mm, with mesh convergence, seeded-imperfection, friction and full-field kinematic checks. The chiral arm angle acts as a mechanism switch: lattices with *θ* = 15–45° deform by node rotation and are auxetic, whereas *θ* = 0° and *θ* ≥ 50° produce stretch-dominated, non-auxetic behavior (full-field ν*_ff_* = +0.11 to +0.83), with the transition between 45° and 50°. The auxetic magnitude forms a plateau of ν*_ff_* = −0.60 to −0.67 across 15–30° at 20% strain and weakens to −0.23 at 45°. These kinematic conclusions are independent of mesh density, friction coefficient and seeded imperfections.

With an intact material model on the baseline mesh, the 15° configuration maximizes quasi-static load-bearing and energy absorption (1452 N/mm effective stiffness, 7.26 kN peak force, 154.5 kN/kg specific load capacity and an upper-bound SEA of 1306 J/kg at 20% strain). Three independent findings nevertheless disqualify this architecture: a collapse-mode bifurcation, in which solutions of the same path obtained with different increment sizes or mesh densities follow two lateral branches separated by 4 mm at essentially identical load, the stiffness peak being an isolated spike between the single-branched 0° and 20° lattices; a maximum principal stress (57.3 MPa) above the data-sheet tensile strength of PA12 already at 10% strain; and a damage-bounded projection anchored on the measured 20% elongation at break that places fracture onset at 3.5–3.6 mm of stroke and reduces its usable SEA to 437–449 J/kg, the 15° lattice being the first to reach the limit for every fracture strain from 0.10 to 0.30 and at every mesh level. By contrast the 30° lattice sustains 9.40 mm and 820 J/kg on the baseline mesh (4.74 mm and 3.44 kN peak force on the finest complete mesh), exceeding its 20°, 25° and 35° neighbors in damage-bounded stroke and energy, and the non-auxetic 0°/90° limits reach 776–832 J/kg.

The 30° architecture therefore emerges as the balanced optimum for printable auxetic absorbers, being the most damage-tolerant member of the 20–30° auxetic plateau and the one furthest from the 15° instability, while the 0°/90° limits are preferable only where the auxetic function is not required. Two findings of the verification program qualify the absolute figures: the response is independent of the friction coefficient, so the response within 20% strain is governed by plastic hinge formation and ligament reorientation rather than by contact; and the reaction forces, energies and fracture-onset strokes decrease by 20–30% on meshes two to nine times finer while the kinematics and the ranking are preserved, so the absolute values are upper estimates and the comparative conclusions are the robust product of the study. All conclusions pertain to the quasi-static crushing of a rate-independent, damage-free, data-sheet-derived constitutive model in the favorable build orientation; the study is purely numerical, and the model is verified but not validated. Mesh-refined re-analysis with a regularized damage model, explicit-dynamics simulation and DIC-instrumented repeated compression experiments on printed 15°, 20° and 30° specimens constitute the pathway from this baseline towards validated, impact-rated deployment.

## Data Availability

The finite element models and post-processed data are available from the corresponding author upon reasonable request.
